# A model for the size distribution of marine microplastics: A statistical mechanics approach

**DOI:** 10.1371/journal.pone.0259781

**Published:** 2021-11-30

**Authors:** Kunihiro Aoki, Ryo Furue

**Affiliations:** Japan Agency for Marine-Earth Science and Technology, Yokohama, Kanagawa, Japan; University of Guam, GUAM

## Abstract

The size distribution of marine microplastics provides a fundamental data source for understanding the dispersal, break down, and biotic impacts of the microplastics in the ocean. The observed size distribution at the sea surface generally shows, from large to small sizes, a gradual increase followed by a rapid decrease. This decrease has led to the hypothesis that the smallest fragments are selectively removed by sinking or biological uptake. Here we propose a new model of size distribution, focusing on the fragmentation of marine plastics. The model is inspired by ideas from statistical mechanics. In this model, the original large plastic piece is broken into smaller pieces once by the application of “energy” or work by waves or other processes, under two assumptions, one that fragmentation into smaller pieces requires larger energy and the other that the occurrence probability of the “energy” exponentially decreases toward larger energy values. Our formula well reproduces observed size distributions over wide size ranges from micro- to mesoplastics. According to this model, the smallest fragments are fewer because large “energy” required to produce such small fragments occurs more rarely.

## Introduction

A large fraction of the estimated billion tonnes of plastic waste that goes into the ocean [1] is found in a fragmented form, “microplastics”, with sizes of less than 5 mm [2] through photodegradation and weathering processes [3–5]. Those microplastics spread globally [6–9], potentially acting as a transport vector of chemical pollutants [10–12] and causing physical and chemical damages on marine organism [13–15]. Recent drift simulations of microplastics calibrated against observed abundance of microplastics have produced global or semi-global maps of estimated microplastics abundance and concentration near the sea surface [6–8]. These simulations will be further used to assess the biological impacts of microplastics.

Such simulations generally assume the size distribution of microplastics and their results would depend on the assumption because sedimentation and biological uptake can depend on size [14, 16–18]. Interestingly, the number of small pieces rapidly decreases toward smaller sizes of *O*(100 μm) in most observations of plastic fragments at the sea surface [19–22]. This feature is puzzling because the number of plastic pieces is expected to increase toward smaller sizes if the pieces keep broken down into smaller and smaller pieces (progressive fragmentation). For example, Cózar et al. [20] indicates that a type of progressive fragmentation leads to a cube law toward smaller sizes. The observed decrease at smaller sizes has generally been hypothesized to be due to selective sinking to depths, sampling error, or to selective ingestion by marine organisms [19–21, 23–27].

The fracture mechanisms of marine plastics, however, are not well known. The power law, which results from scale-invariant fracture processes, is often invoked to explain observed size distributions of plastics. It tends to fit well observed size distributions at larger sizes [20]. The power law can be derived, for example, from collision cascade among objects, as often applied to the fragmentation of asteroids [28–30], which does not include any decrease toward smaller sizes unless or until other processes than fragmentation start to dominate. Somewhat different fragmentation processes lead to a log-normal distribution [31–33], which is premised on the size reduction rate following the Normal distribution and has been applied to the fragmentation of sand grains, mastication, and the fracturing of thin glass rods [34–36], but not to oceanic microplastics to our knowledge. The log-normal distribution has a peak skewed toward smaller sizes and is similar to observed size distributions of fragments at smaller sizes but tends to deviate from observed distribution at larger sizes, where the power law tends to fit better [20]. To the best of our knowledge, only the Weibull distribution [37, 38] is similar to observed size distributions across the microplastics and mesoplastics ranges (fragments larger than 5 mm), at least in Cózar et al.’s study [20]. The Weibull distribution as applied to the size distributions of various types of particles is largely empirical but with an interpretation as resulting from the branching tree of cracks [38].

The microplastics are thought to result from fragmentation by mechanical forces associated with waves or winds [3–5], but linkage between the microplastics and waves or winds has not been formulated in a physical manner. In this paper, assuming that “energy” needed to break down the plastic pieces acts as a constraint, we propose a new approach to modeling the fragmentation of marine plastics in relation to potential mechanical forces exerted on the plastic pieces borrowing ideas from statistical mechanics. As we shall see, under the assumption that the probability of the “energy” obeys an exponential distribution, the model reproduces a size distribution having a log-normal-like shape toward the small-size end and a power-law shape toward the large-size end. Both log-normal and power-law distributions have been commonly identified in observations from the surface to the mesopelagic levels in the ocean and on a beach [8, 19–22, 24, 39, 40]. We will further discuss a potential explanation of the observed size distribution under the presence of deep intrusion or sampling error.

## Fracture model

We assume that plastic waste first becomes fragile on beaches because of ultraviolet light and other weathering factors and then is broken up on beaches into microplastic pieces by the action of waves, winds, and other forces before being washed off into the ocean. Accordingly, the amount (mass) of microplastics which is produced on beaches will be determined by these processes. The fraction of microplastics that goes into the ocean may depend on waves and other weather factors. The microplastics will then be diluted in the ocean by mixing due to turbulence. These are the microplastics which observations sample. In the following we build a simple, idealized statistical model that determines the size distribution of the microplastics produced on the beaches. The model considers the fragmentation of given plastic mass and is naturally mass-conserving.

We offer the following simple physical model only as a representation of complicated fragmentation processes such as one-time crush and slow weathering. We do not claim that the following are exactly the underlying processes that lead to the observed size distribution. This model is originally intended to explain microplastics larger than *O*(100 μm). It will be later extended for finer microplastics (see the Discussion section and S4 Appendix in S1 File). In this section, we only show an outline of the derivation. Details are found in “Details of fracture model” of the Materials and Methods section.

**Fig 1:**
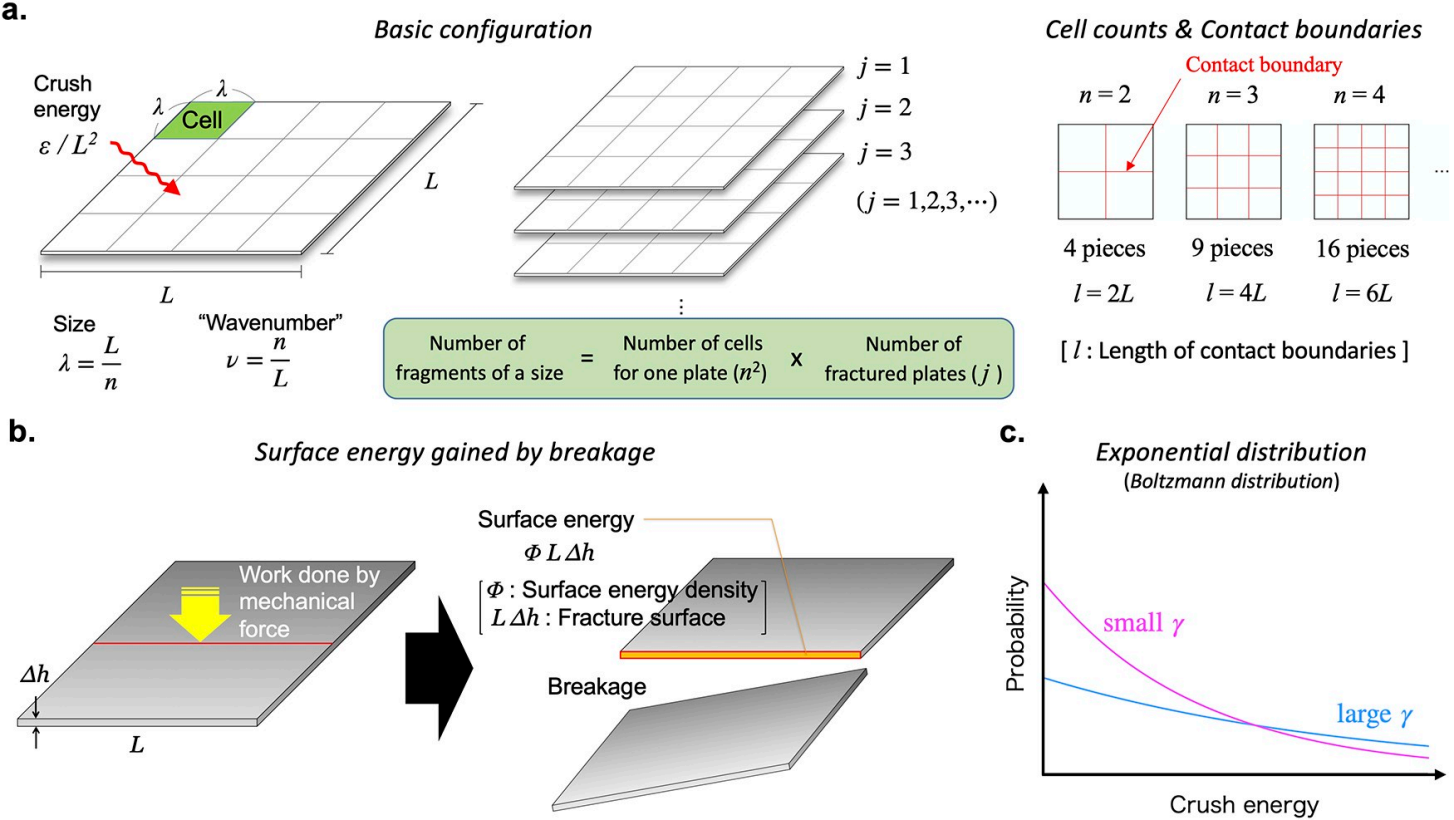
Schematic illustration of model configuration. **a**. Schematic representation of our fracture model. An idealized plastic plate with a size of *L* × *L* and a thickness of Δ*h* is broken into *n* × *n* equal-sized pieces. The size of each broken piece is λ = *L*/*n* (left). The total length of the contact boundaries (red lines) is obviously *l* ≡ 2(*n* − 1)*L* (right). We sum up energy needed to break *j* plates into λ-sized pieces and denote it as *ε* (middle). **b**. Schematic representation of breakage. Energy needed to break up the plate is proportional to the cross-sectional area of the contact boundary and hence to the length of the fracture. **c**. Occurrence probability of crush energy governed by the Boltzmann distribution, *p*(*ε*) = *e*^−*ε*/*γ*^/*γ*, which exponentially decreases for larger crush energy *ε*. The e-folding scale is *γ* and hence the probability of larger crush energy becomes larger as *γ* increases.

The model assumes that the original plastic piece is a square plate with a size of *L* × *L* and a uniform thickness of Δ*h*. This plate is broken into *n* × *n* equal-sized cells (Fig 1a). In this case, the size of each broken piece is λ = *L*/*n*. The number of the fragments of this particular size is given by *n*^2^ multiplied by the number of the original plates (Fig 1a).

The fragmentation is assumed to be caused by “crush energy” that represents any mechanical forces such as winds and waves. In general, the energy required to fracture a brittle material can be evaluated by the surface energy (defined per unit area) multiplied by the area of the newly created surfaces (Griffith’s theory [41, 42]) (Fig 1b). Exploiting this fact, we define the crush energy here as the total surface energy of the broken pieces for each *n*. Highly plastic or nonlinear fracture processes involve other types of energy than surface energy [43–45], but we assume that breakage happens only after sufficient degradation and therefore that the plastic material is fully brittle [4, 5], in which case, only surface energy needs to be considered. For a uniform surface energy density over the new surfaces, the total surface energy of broken pieces is proportional to the total length of the contact boundaries (Red lines in the right panel of Fig 1a) since the plate is assumed to have a uniform thickness. Because the total length of the contact boundaries increases linearly with *n* (Fig 1a, right), the required crush energy is proportional to the total length of all contact boundaries. This means that to produce smaller pieces requires larger energy, which ultimately limits the number of small fragments as shall be seen below. This is the most significant new element of our model.

Plastic pieces are fragmented by the action of waves, winds, or sand under various conditions, for example, on a hard reef or on soft sand, and therefore energy exerted on the plastic pieces is considered random. Unless each of these processes is modeled in detail, however, it is impossible to calculate the probability distribution of the energy from first principles. Nor is it possible to infer the probability distribution from field measurements at present. To make progress, we assume that an exponential distribution, *p*(*ε*) ∝ *e*^−*ε*/*γ*^, governs the occurrence probability of crush energy *ε* (Fig 1c). This distribution is called “Boltzmann distribution” in the field of the statistical mechanics, which is often assumed *by default* when details of the underlying stochastic processes are not known and the distribution is known to be applicable to a wide variety of situations [46–49]. This generality comes from the fact that it is the distribution that maximizes the entropy under fixed total energy [50]; that is, it is the most probable energy distribution under fixed total energy. We note that the long-term statistics of the coastal wave height generally shows that a larger wave height occurs with a smaller frequency [51–53], qualitatively consistent with the exponential distribution.

According to this probability distribution, a crush event with a large energy value is less frequent, consistent with common expectation. The factor *γ* may be regarded as a representative energy level of *the environment,* which represents the aggregate impacts of the combination of weather conditions (winds, waves, etc.) and the background conditions (hard or soft surfaces, etc.).

The number of the fragments of a particular size is *n*^2^*j* for a single crush event (Fig 1a). Since crush events occur randomly, the size spectrum is the expected value of *n*^2^*j* as a funcrtion of λ. Calculating the expected value with use of *n* = *L*/λ, we arrive at the size spectrum (see “Details of fracture model” of the Materials and methods section)

$$\begin{array}{c} S\left(\lambda \right)d\lambda =\frac{A}{\lambda^{4}}\frac{1}{e^{b/\lambda \gamma }-1}d\lambda , \end{array}$$ (1)

where *A* is an arbitrary positive constant, which is adjusted by the amount of the total mass of plastics to be fractured.

**Fig 2:**
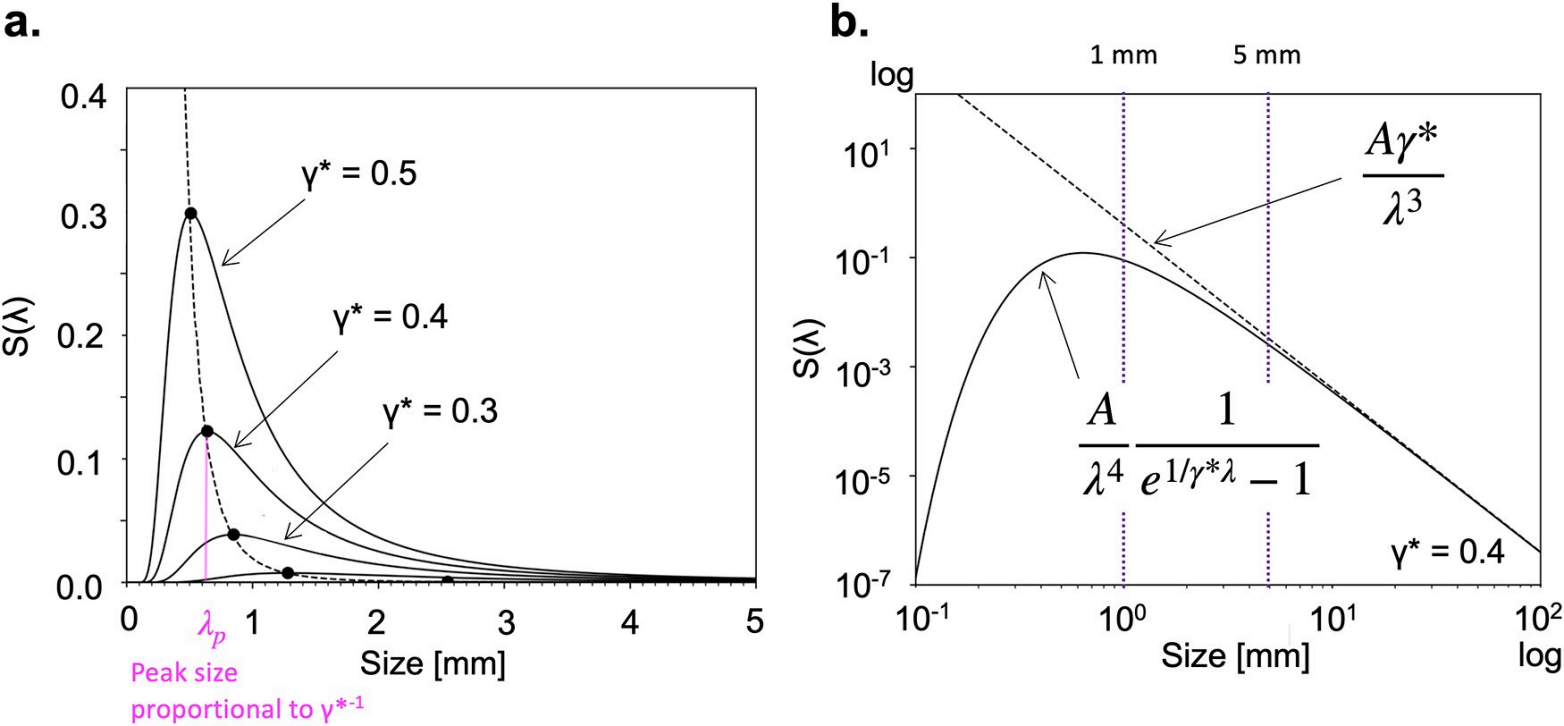
Theoretical size spectrum. **a**. size distributions expected from (1) for different values of γ* under a fixed *A* (= 1.0). The dashed curve connects the peaks of the size distributions. **b**. a log-log plot of the same distribution for γ* = 0.4 and *A* = 1.0 (solid curve). The dashed line denotes the power law Aγ*/λ^3^, which the size-distribution curve asymptotes to for large λ.

This size distribution has a shape skewed to smaller sizes similarly to the observed size distribution of the microplastics, as shown in Fig 2. For a fixed *A*, the size distribution is controlled by *γ* and *b*. The factor *b* depends on the plastic material and the size of the original plate. The increase of *γ* and the decrease of *b*, however, have the same effect on controlling the size distribution and hence we introduce a new parameter γ* ≡ *γ*/*b*. This parameter measures the strength of the mean environmental energy against the strength of the plastic material and its inverse provides a characteristic size such that the peak size, λ_*p*_, is given by λ_*p*_ ≃ 0.255γ*^−1^. As this parameter increases, thus, the size at which the maximum of the size distribution occurs decreases like γ*^−1^ and the corresponding maximum value increases like γ*^4^ (Fig 2a). Furthermore, in the large size limit, i.e., λγ* ≫ 1, this size distribution asymptotes to *S*(λ)*d*λ ∼ Aγ*/λ^3^*d*λ (Fig 2b), consistent with the cube power law observed in the mesoplastic range [20].

The total abundance and mass of the fragments for λ < Λ (≤*L*) are obtained, respectively, as

$$\begin{array}{cc} N & \equiv \int_{0}^{\Lambda }S\left(\lambda \right)d\lambda \approx \int_{0}^{\infty }S\left(\lambda \right)d\lambda =\sigma A{\gamma^{*}}^{3},\\ M & \equiv \int_{0}^{\Lambda }\;\rho \Delta h\lambda^{2}S\left(\lambda \right)d\lambda =\rho \Delta hA\gamma^{*}\text{ln}\left(1-e^{-1/\gamma^{*}\;\Lambda }\right)\approx \rho \Delta hA\gamma^{*}\text{ln}\left(\gamma^{*}\Lambda \right), \end{array}$$ (2)

where *σ* ≡ 2.404 (S1 Appendix in S1 File), *ρ* is the plastic density, and Λ is the maximum size of plastic fragments. Both of the above approximations are for γ* Λ ≫ 1, that is, Λ being sufficiently larger than the characteristic size λ_*p*_. See S1 Appendix in S1 File for details.

We fit our model (1) to an observed size spectrum by adjusting *A* and γ*. Observed size distributions are presented in various ways: as the number per unit volume of sampled sea water, as the number per unit surface area of the ocean, as the raw number of collected fragments, etc. Some studies normalize the spectrum so that ∫*S*(λ)*d*λ = 1 [24, 25, 39]. However, note that the size spectrum (1) can take any of these forms by adjusting *A* and different representations of the same spectra do not affect our analysis because the differences are absorbed into *A*.

**Fig 3:**
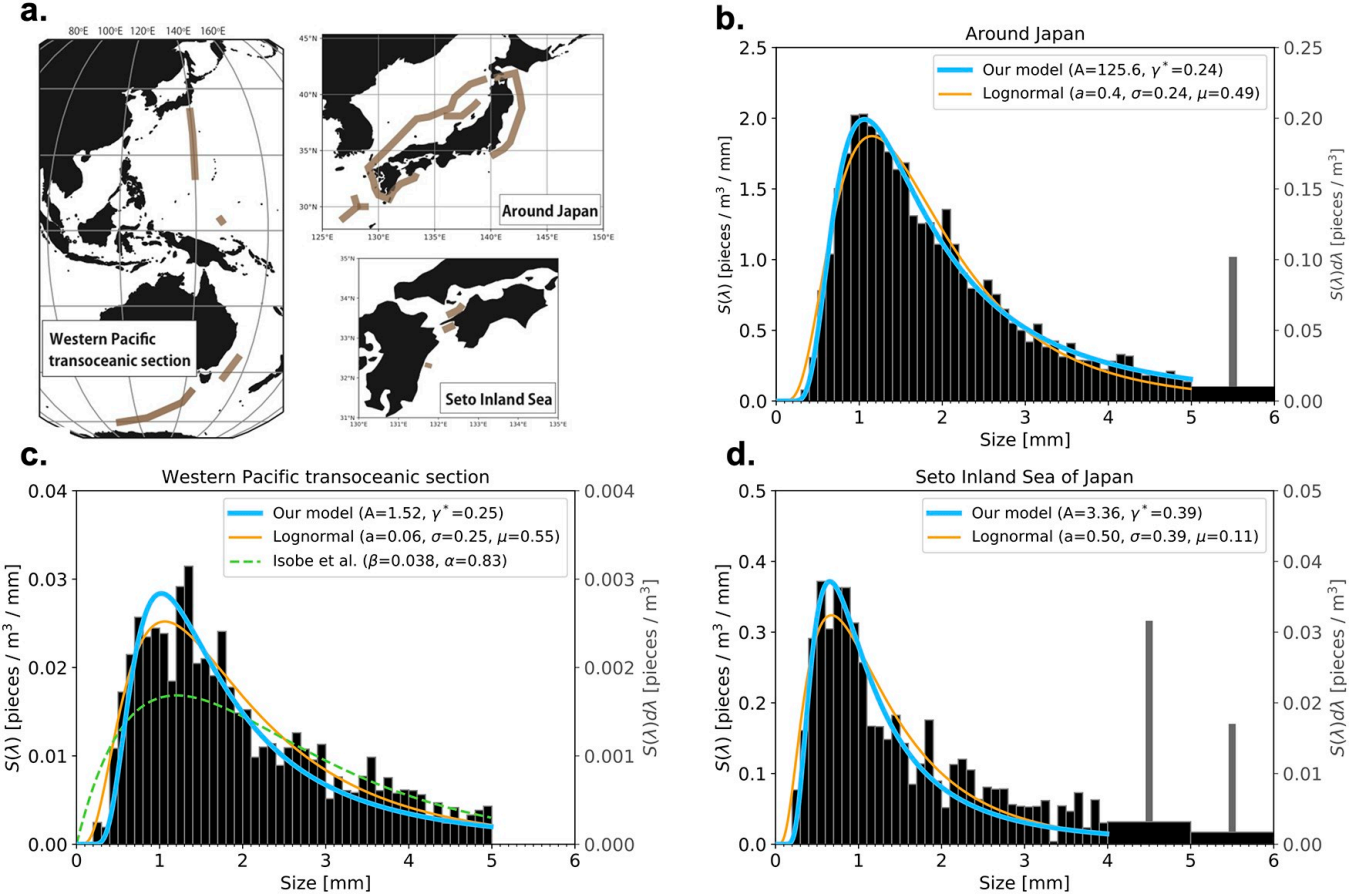
Comparison of theoretical size spectrum with the observed size distributions of Isobe et al. [8, 19, 21]. **a**. Schematic map of observation stations in Isobe et al. [8, 19, 21]. See their original papers for the detailed locations. **b–d**. Theoretical and observed size spectral densities of microplasitcs in the area surrounding Japan, along a Western-Pacific transoceanic section, and in the Seto Inland Sea. The black bars display the observed size spectral density (left axis), and gray bars are the original histogram (right axis) taken from Isobe at al. In the range λ < 5 mm, the black and gray rectangles perfectly coincide. See “Observed data in Isobe et al” in the Method section for the detailed method of the conversion from the histogram to the spectrum. The blue and orange curves denote our model and the lognormal distribution, respectively. The parameter *A* is dimensionless (×10^−9^) in this case. The green dashed curve in **c** denotes the empirical curve *β*λ*e*^−*α*λ^ of Isobe et al [8]. The map in **a** is created using a Python package Cartopy [54] (version 0.18) and Adobe Illustrator CS6 (version 691; https://www.adobe.com).

We begin by comparing the present theory with the observed size distribution obtained by Isobe et al [21]. This size distribution is based on the collection of plastic fragments sampled around Japan (Fig 3a); this is the largest collection with the highest size resolution to date. For a precise comparison, we have converted the original size histogram into spectral density. Details are shown in “Observed data in Isobe et al” of the Materials and methods section.

### Application to observed data

Theoretical curve is fitted to the observed size spectrum by adjusting the parameters *A* and γ* by a least-squares method over λ < 5 mm. The theoretical curve fits the observed size distribution well over the whole microplastic range (Fig 3b) with the relative error, Err = 7%. (The relative error is defined by the norm of difference between the theoretical and observed size distributions divided by the norm of the observed size distribution.) For comparison, we also plot a lognormal distribution, *a*LN(*μ*, *σ*^2^), with an amplification or normalization factor *a*, where the parameters *a*, *μ*, and *σ* are determined by the same least-squares method. The lognormal distribution is not much different from our model in this size range.

Our model also agrees well with the observed size distributions in the Western-Pacific transoceanic section (Err = 22%) and the Seto Inland Sea (Err = 21%) (Fig 3c and 3d) with somewhat larger error than around Japan. The observed spectra are not as smooth as that around Japan, suggesting that the samples are not sufficient to give smooth distribution and this may be the reason for the larger error. Also, the peak is located at a smaller size in the Seto Inland Sea than in the other two regions; this shift is reflected in a larger value of γ*. The lognormal distribution is somewhat more different from our model in these two regions (Fig 3c and 3d) than in the area surrounding Japan (Fig 3b). The empirical fitting curve used in Isobe et al [8] has larger error, despite having the same number of parameters as ours (Fig 3c).

**Fig 4:**
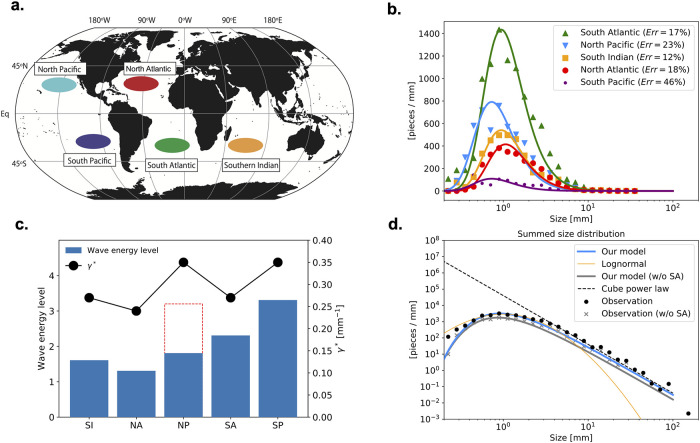
Application of theoretical model to Cózar et al. [20]’s observational data and comparison between γ* and wave energy. **a**. Schematic map of observation stations in Cózar et al. [20]. See the original paper for the detailed locations. **b**. Theoretical (curves) and observed (symbols) size spectral density of all samples for each accumulation zone. The observed data are same as those in their S6 Fig, except that the values are expressed as a spectral density (see “Observed data in Cózar et al” in the Materials and methods section). **c**. Expected wave energy levels (no units) at source regions (rectangles) and γ* (dots) for accumulation zones. The accumulation zones are denoted by abbreviations such as SI for South Indian Ocean. The red dashed rectangle shows the wave energy level for the North Pacific Ocean in the case where the contribution from China is removed. See “Wave energy in source region” in the Materials and methods section for details. **d**. Sum of the size spectral densities plotted in **b** over the basins for observation (black dots) and our model (blue curve). The orange curve and black dashed line denote lognormal distribution and a cube power law, respectively. The observed data and cube power law line are the same as those in Cózar et al’s S10 Fig. The gray dots and curve are the same as the black dots and blue curve, respectively, except that the South Atlantic data is excluded for the sizes less than 30 mm (the digitizer does not recognize the small spectral values above 30 mm for the South Atlantic data in Cózar et al’s figure). The scale of each axis on panels **b** and **d** follows that of Cózar et al’s S6 and S10 Figs, respectively, except that the vertical scale on panel **b** is shown as spectral density. The map in **a** is created using the same softwares as in Fig 3a.

We next compare our model with another set of observations summarized by Cózar et al [20] (Fig 4a) based on samples collected in the accumulation zones (“garbage patches”) over the globe, where microplastics originating from coastal areas tend to converge by ocean currents [55, 56]. Cózar et al’s method of sampling plastic fragments is similar to Isobe et al’s [8, 19, 21], but their size distributions include more size bins in the mesoplastic range than Isobe et al’s (See “Observed data in Có” of the Materials and methods section). Our model generally reproduces well the observed size distributions although it has large relative error in the South Pacific Ocean (Fig 4b), where the least number of samples were collected in Cózar et al.’s study and the spectrum is the least smooth (barely visible in the plot), suggesting that the number of samples is not sufficient. The unweighted sum of the five size distributions is shown in Fig 4d in a log-log form. Importantly, the model reproduces the cube power law toward the mesoplastic range (Blue curve in Fig 4d) as found by Cózar et al. The theoretical curve is not particularly good in the smallest size range, but this discrepancy reduces (gray curve and symbols in Fig 4d) when we exclude the South Atlantic Ocean, which has large difference from the model in the sizes smaller than 0.5 mm (green curve and symbols in Fig 4b). If the plastic pieces sampled in the South Atlantic came from different regions with a wide variety of γ* values, this discrepancy may be explained (Figs. B and C in S3 Appendix in S1 File). In contrast, the lognormal distribution does not follow the cube power law in the large size range (orange curve in Fig 4d) and this discrepancy exists also in the case without the South Atlantic data (not shown). Despite having one more adjustable parameter, the log-normal distribution fit the data less well than our model.

The fitting of our model to observations gives a geographical distribution of γ*. The γ* value for Cózar et al’s observation is largest in the Pacific Oceans, smallest in the North Atlantic, and inbetween elsewhere (Fig 4c). Given that the plastics are likely to be fragmented on beaches possibly by ocean waves [4], the value of γ* would represent wave energy on beaches where plastic waste is littered or comes ashore before fragmented there and washed away into the ocean as micro- or mesoplastics. A scenario-based numerical experiment (see “Wave energy in source region” of the Materials and methods section) suggests that the coastal area located along the western boundary of each basin is likely the major source region for the plastics in each accumulation zone, except that a large fraction of the plastics in the South Indian Ocean comes from South East Asia [55]. Compared with a map of categorized wave energy levels along the world coastlines [57], the wave energy level in the potential source regions for a basin (Table 1) appears correlated with the γ* value for the accumulation zone of the basin in the emission scenario considering impervious area of land (Fig 4c; the results for the other scenario are found in Table 1 and S1 Fig in S1 File). The only exception is the North Pacific: the North Pacific accumulation zone has a large γ* value in spite of the relatively small wave energy level in the South China Sea, likely the major source region for the North Pacific. In fact, a large fraction of the plastic emission from the East China Sea is likely to come ashore on the southern coast of Japan [55], where the wave energy level is large (Table 1). If the majority of plastic waste is fragmented there, therefore, the large γ* value for the North Pacific may be explained (Fig 4c).

**Table 1:** Expected wave energy levels (based on Fairley et al’s [57] map) at source regions and plastic emissions (percentage) that contribute to the five major oceanic accumulation zones based on the two terrestrial release scenarios (ISA scenario and PD scenario) from Lebreton et al. [55]. The categorization of the source regions is from Lebreton et al’s supplementary material. The values for the ISA and PD scenarios are indicated in the left and right sub-columns of each column, respectively. See the Methods section for the details.

Sources	Wave energy level		Accumulation zones									
			SIO		NA		NP		SA		SP	
Aus	5	5	0.089	0.185			0.000	0.000	0.005	0.009	9.502	15.976
NZ	5	5	0.017	0.002			0.000		0.035	0.006	31.211	4.213
South Am wst	3	3	0.007	0.001			0.049	0.084	0.020	0.001	20.376	35.645
South Am est	2	2	7.530	4.435	5.317	6.570			58.530	79.591	1.538	0.986
Cent Am est	2	2			13.294	20.841						
North Am est	1	2			50.281	44.931						
Cent Am wst	4	4	0.006	0.000	0.018		3.793	0.589			5.532	1.457
North Am wst	4	4	0.003	0.003			8.822	7.337			0.056	0.062
Canada wst	5	5	0.000	0.000	0.151		0.569	0.205				
Africa est	2	2	25.872	7.434				0.000	5.982	3.861	9.422	2.873
Africa wst	3	6	2.779	0.676	4.194	5.076			33.906	13.386	0.412	0.148
Africa Nth	3.5	3.5			1.908	5.672				0.001		
Arabia	1	1	0.410	1.001					0.017	0.088	0.050	0.106
Med	1	1										
Asia SE	1	1	10.748	5.509			1.131	0.695	0.155	0.163	4.142	2.218
Indonesia	1	1	27.645	60.278			1.603	5.094	0.381	1.826	13.495	30.801
China	1	2	7.243	11.588			65.784	58.036	0.076	0.227	2.456	4.114
Russia est			0.024	0.007	0.134		7.385	0.742			0.007	0.002
Black Sea												
North Europe	1	6			24.004	16.836		0.000	0.000			
Canada est					0.555							
GreenLnd												
Russia wst					0.144	0.075						
India wst	2	2	5.136	3.311					0.238	0.306	0.537	0.473
India est	2	2	12.294	4.991				0.652	0.652	0.523	1.165	0.655
Japan	5	5	0.197	0.578			10.863	27.217	0.010	0.012	0.099	0.271
**Expected wave energy level**			1.6	1.4	1.3	3.0	1.8	2.9	2.3	2.5	3.3	2.7
			Without China →				3.2	4.1				
**Expected wave energy level (Ave.)**			1.5		2.1		2.3		2.4		3.0	
			Without China →				3.7					

The interpretation of γ* for Isobe et al’s observations is less straightforward because the source region is less clear. The relatively low value of γ* for the area surrounding Japan (Fig 3b and S1 Table in S1 File) may be due to the low wave energy in the East China Sea, which may be the source region of the microplastics [55]. In contrast, there are clearly multiple source regions contributing to the Western Pacific transoceanic section and it is possible that the distribution is a superposition of distributions with different γ* values (Fig B in S3 Appendix in S1 File). It is interesting that the Seto Inland Sea has a very large γ* (Fig 3d and S1 Table in S1 File), which may be due to some local conditions or to the conditions of some remote locations where the microplastics originate. It is indeed possible that some microplastics in this region originate from the Philippine Sea, where wave energy is large [57], as previous studies indicate that some waters from the Philippine Sea are transported into the Seto Inland Sea through the western boundary current [58–60].

## Discussion

In summary, we derive a theoretical size distribution of micro- and meso-plastics using a statistical mechanical approach. It assumes that larger “energy” is required to break down the original plastic piece into smaller fragments and that this energy follows the Boltzmann distribution. This model well reproduces observed size distributions from the micro- to meso-plastics. In particular, it naturally explains the rapid decrease toward smaller sizes without invoking a removal of smaller fragments. This model is highly idealized, and extending this model for more realistic fragmentation processes that may be involved is a future study.

As pointed out above, Cózar et al’s South Atlantic size distribution deviates from our theoretical curve more than the other size distributions in the same dataset. S3 Appendix in S1 File considers theoretical size distributions that would result if the sample is a mixture of plastic pieces originating from various source regions with different values of γ*, assuming that each source contributes the same number of plastic fragments for simplicity. The resultant superposition deviates from the single-source size distribution in a similar way that Cózar et al’s South Atlantic size distribution deviates from the single-source theoretical distribution (Fig C in S3 Appendix in S1 File). Interestingly, the value of 1/γ* that best fits the mixture is close to the average of 1/γ* values of the origins in this simple example (See “Size distribution” in S3 Appendix in S1 File).

Similarly, a mixture of plastic fragments produced in various conditions may need to be considered for interpreting the observed total plastic mass. Isobe et al [8] show that the total mass of the plastics for sizes below 5 mm is about 0.3 mg m^−3^ for their samples in the Western-Pacific transoceanic surveys assuming that *ρ* = 1000 kg m^−3^. To obtain this value from our *M* formula (2) with the optimal values of *A* and γ* for their dataset and with Λ = 5 mm, we arrive at a value Δ*h* ≈ 1 mm. Obviously this value of Δ*h* is too large for the fragmentation model presented in this study because fragments smaller than Δ*h* cannot be produced by the two-dimensional fragmentation of a plate with a thickness of Δ*h*. This problem may be resolved if the value of Δ*h* obtained like this is in fact an average of the various thicknesses of the original plastic plates (see “Total mass” in S3 Appendix in S1 File).

We have attempted to explain the observed size distributions at the surface by fragmentation alone, but in reality, a large fraction of the fragments are likely to be missing from observations. For example, deep intrusion is thought to be necessary to explain the amount of floating plastics [8, 61], which is far less than estimates of terrestrial plastic emission [6, 20]. Biofouling, accumulation of organic matter on the surfaces of plastic fragments, is probably the primary mechanism for microplastics to lose buoyancy and sink to depths. This mechanism is more effective for smaller microplastics and therefore can affect the size distribution of microplastics at the sea surface [62–64]. Sampling error can also affect observed size distributions. Some studies report that the rapid decrease toward the small size common to most size distributions at the surface may be an artifact attributable to the method of collection or size detection [24–27]. If so many smaller microplastics are actually missing from observations, either by sinking or by escaping the nets, as to significantly alter the observed size spectra, the γ* values we have obtained by fitting are obviously underestimated, supposing, of course, that our theory is correct.

Further, secondary fragmentation may produce finer fragments. Since the smaller microplastics are closer to three-dimensional shapes than to a plate, their fracture would be a three-dimensional process. The 3-dimensional version of our model (Fig. D in S4 Appendix in S1 File) can be similarly constructed to the 2-dimensional version (Fig 1a). The result is such that the model spectrum (1) gets one more factor of λ^−1^ and γ* = *γ*/(3*L*′^3^*ϕ*), where *L*′ is the size of the original microplastic cube. The peak size is at λ_*p*_ = 0.201γ*^−1^. See S4 Appendix in S1 File for a complete derivation. The theoretical curves of the 3-dimensional model are found to be in good agreement with the observed size spectra of the fine microplastics with scales of *O*(10 μm) collected near the sea surface [24, 39], in the upper ocean from the surface to the mesopelagic layer [40], and on a beach [22] (Fig. E in S4 Appendix in S1 File). Those theoretical curves are characterized by γ* values of 1.68–14.01 mm^−1^, which are 10–100 times larger than the two-dimensional γ* values of 0.24–0.39 mm^−1^ for Isobe et al.’s [8, 19, 21] and Cózar et al.’s [20] (Figs 3 and 4). Note that, however, the energy requirement for the fragmentation is given by *γ*. As an order-of-magnitude calculation, the value of *γ* = 3*L*′^3^ϕγ* from the three-dimensional γ* value is *O*(10^3^) smaller than the value of *γ* = 2*L*^2^ Δhϕγ* from the two-dimensional γ* value under the reasonable assumptions that *L* = 100 mm and Δ*h* = *L*′ = 1 mm and that the surface energy density *ϕ* is the same. This small *γ* value comes from the smallness of the initial size, *L*′, which we assume that the three-dimensional fragmentation starts from. Plastics with smaller initial sizes can be fragmented by smaller energy since the surface energy decreases with the initial size (see Fig 1b in this text and Fig D in S4 Appendix in S1 File).

Accordingly, the fate of the marine plastics may be as follows. Original plastic pieces deposited at a beach are fragmented down to millimeter size by weather phenomena such as waves. The two-dimensional version of our model intends to predict the spectrum of these fragments. Those microplastics are further fragmented into finer pieces by slow weathering, grinding in sand, or other processes. The three-dimensional version of our model may also explain the resultant size spectrum of the finer fragments. Both types of microplastics are washed off into the ocean. Given that a smaller particles are susceptible to the vertical mixing [19, 23, 24, 65], those finer microplastics may be spread over the mixed layer or deeper, while the larger ones may tend to stay at the sea surface. This scenario based on our fracture models has no contradiction to the observed evidence on the spatially varying size distribution of the microplastics so far.

The reduction in the particle count at smallest sizes would be a physically reasonable feature when available fracture energy is limited. Indeed, the general grinding needs larger energy to create smaller particles [66, 67], and the size distribution resulting from a progressive grinding process has this feature [68]. The power law, which the literature often invokes to explain size distributions, is usually ascribed to some progressive fragmentation processes. Such processes may also result in a decrease at small sizes if a limitation of energy is introduced as it is to our model. When the observed spectrum drops at smaller sizes, the lognormal distribution is also often invoked and contrasted to the power law in the literature on experiments for plastic fragmentation [69, 70]. Quantitatively, the lognormal distribution does not much differ from our model spectrum (Figs 3 and 4) although it does not agree with the power law in the larger-size range (Fig 4d). Distributions which were compared to the lognormal distribution in the literature may be explained by similar mechanisms to our model.

The present model is premised on the fragmentation of a plate into equal-sized cells with a regular square shape, which provides the inverse proportionality in the crush energy to the size (See section). However, this square lattice is merely a simple device to relate the total surface energy (the work required to create new surfaces) with the size and the number of fragments, but this regularity is not necessary. Indeed, the same relationship between the crush energy and the size holds for irregular cells given by a Voronoi tessellation [71] (S5 Appendix in S1 File), which is often used for modeling pervasive fracture such that an impact causes propagation of multiple cracks and collapse [72–76]. The pervasive fracture caused by an instantaneous impact generally can yield the fragments with various sizes. Our modeled size spectrum may be regarded as an ensemble of the fragment counts for each size for multiple pervasive fracture events with a nearly uniform impact.

The scale invariant approach treating fractality in fracture is often used to describe the fragmentation process of materials into isolated pieces and can explain power law size distributions in fragment counts observed in the breakage of the materials such as concrete, glass, and ice in nature [77–79]. In these analyses, consistent with Griffith’s theory of crack extension, the energy consumption to create fragments are represented by surface energy, which is proportional to the surface area of the fractures [80–84]. Our model shares this property. An essential difference between the two approaches is that our model limits the available energy by introducing occurrence probability of crush energy. As a result, our distribution takes a qualitatively similar form to the log-normal distribution in the smallest-size region and reduces to a power law in the large-size limit (See Fracture model section). The latter limit is given by λγ* ≫ 1, which is equivalent to the large γ* limit; that is, when γ* is large, the power law region extends leftward (toward smaller sizes in the size spectrum). Unlike in fractal processes, the exponent in the power law size distribution is an integer number in our model, which is a consequence of crush energy (total surface energy from a single plastic plate) being inversely proportional to the size of the fragments. In the above argument, we have shown that this proportionality holds even for the irregular fragment cells following a Voronoi tessellation but we have not permitted surface fractality. Further generalization of our model is needed to cover a wide range of fractal processes.

The present fracture model may open paths toward developing sophisticated numerical simulations for predicting the production and dispersion of the microplastics. There have been numerical simulations to evaluate the spreading microplastics in the ocean [6–9, 85]. In such simulations, virtual parcels representing a mass of plastic pieces are released at source regions and advected by ocean currents. The amount of plastics released has to be either assumed or calibrated so that the resultant mass distribution matches observations. The size distribution, if that information is necessary, is usually assumed in an ad-hoc manner or calibrated against observations. Given that the microplastics are likely to originate from the weathering of plastic litter on beaches [3–5], their size distribution would depend on weather and wave conditions that would be different for each beach. Our size distribution model may be used to estimate the initial size distribution of plastic fragments by parameterizing γ* at the beaches as a function of weather conditions such as winds and waves.

## Materials and methods

### Details of fracture model

In the Fracture model section, we only outlined the derivation of our model spectrum and focused on its interpretation; here we show full details of the derivation. Our model is derived in analogy with black body radiation; see S2 Appendix in S1 File for the analogy.

In this model, we consider that a square plastic plate with a size of *L* × *L* and a uniform thickness of Δ*h* is broken into *n* × *n* equal-sized cells (Fig 1a). The size of each broken piece is given by λ = *L*/*n*; we also define an associated “wavenumber” *ν* ≡ λ^−1^ = *n*/*L* for convenience.

In general, the minimal energy required to fracture a lump of solid is given by the surface energy [86], which is proportional to the area of the newly created surface. This area in the present model is proportional to the total length of the contact boundaries (Fig 1a), which is related to *n*, like 2*L*(*n* − 1) ≈ 2*Ln*. With the aid of *ν* = *n*/*L*, the crush energy for a plate can therefore be written as *bν* with a constant *b* ≡ 2*L*^2^Δ*hϕ*, where *ϕ* is a uniform surface energy density, and hence the crush energy for *j* plates can be written as

$$\begin{array}{c} \varepsilon \equiv jb\nu . \end{array}$$ (3)

We assume that the occurrence probability of crush energy is governed by the Boltzmann distribution characterized by *γ*:

$$\begin{array}{c} p\left(\varepsilon \right)\propto e^{-\varepsilon /\gamma }, \end{array}$$ (4)

which states that a crush event with a large energy value is less frequent, consistent with common expectation. In statistical mechanics, *γ* is temperature in heat bath times the Boltzmann constant (Fig A in S2 Appendix in S1 File). This is the analogy to the “environment” for microplasitics.

From (3), the expected value of the crush energy as a function of *ν*, denoted by 〈*ε*〉_*ν*_, is

$${\left\langle \varepsilon \right\rangle }_{\nu }=\sum_{j=0}^{\infty }jb\nu \;p\left(jb\nu \right)=b\nu {\left\langle j\right\rangle }_{\nu },$$

and from (4),

$$\begin{array}{c} {\left\langle j\right\rangle }_{\nu }=\frac{\sum_{j=0}^{\infty }je^{-jb\nu /\gamma }}{\sum_{j=0}^{\infty }e^{-jb\nu /\gamma }}=\frac{1}{e^{b\nu /\gamma }-1}, \end{array}$$ (5)

which is the expected value of the number of fractured plates for each *ν*. In deriving (5), we used the geometric series such that $\Sigma_{j=0}^{\infty }ar^{j}=a/\left(1-r\right)$, which holds for any *a* and any *r* ≠ 1. This formula is called the Bose distribution [87]. Since the number of the fragments of a particular size is *n*^2^*j* (Fig 1a) and *n* = *Lν* by definition, the expected number of fragments is *P*(*ν*) ∝ *n*^2^〈*j*〉_*ν*_ ∝ *ν*^2^〈*j*〉_*ν*_ and therefore,

$$\begin{array}{c} P\left(\nu \right)d\nu =A\nu^{2}\frac{1}{e^{b\nu /\gamma }-1}d\nu , \end{array}$$ (6)

or converting from *ν* to λ, we arrive at the size spectrum we already presented in the Fracture model section

$$\begin{array}{c} S\left(\lambda \right)d\lambda =\frac{A}{\lambda^{4}}\frac{1}{e^{b/\lambda \gamma }-1}d\lambda , \end{array}$$ (1)

where *A* is an arbitrary positive constant. Eqs 6 or (1) is *formally* analogous to the Planck’s formula of blackbody radiation (See S2 Appendix in S1 File).

### Observed data in Isobe et al

The observed size distributions in Isobe et al are from surveys around Japan [21], in a western Pacific transoceanic section [8], and in Seto Inland Sea [19] (Fig 3a). The first set of samples is from 56 stations around Japan during the period of July 17 through September 2, 2014 [21]. The second is a transoceanic survey at 38 stations across a meridional transect from the Southern Ocean to Japan during 2016 [8]; the stations in Southern Ocean and the other ones were occupied from January 30 to February 4 and from February 12 to March 2, respectively. The third dataset is collected at 15 stations in the Seto Inland Sea of Japan in May–September from 2010 to 2012 [19].

For all surveys, neuston nets with mouth, length, and mesh sizes of 0.75 × 0.75 m^2^, 3 m, 0.35 mm, respectively, were used to sample small plastic fragments. The nets were towed near the sea surface around each station for 20 min at a constant speed of 2–3 knots (1–1.5 m/s). The numbers of fragments are counted for each size bin with a bin width of 0.1 mm for microplastics and 1 mm for mesoplastics (defined to be >5 mm) between 5 and 10 mm, except for the surveys in the Seto Inland Sea, in which the bin width becomes wider beyond the size of 4 mm. The size of a fragment is defined by the longest dimension of its irregular shape. The concentration of the fragments (pieces per unit volume of sea water) within each size bin were calculated by dividing the number of fragments by the water volume measured by the flow meter at each sampling station. This concentration binned according to size is the observational data used in the present study. To obtain the numbers, we have digitized the plots of Isobe et al’s using WebPlotDigitizer version 4.3 (https://automeris.io/WebPlotDigitizer/). The original size distributions in the Seto Inland Sea are presented separately for four different areas [19], but they are averaged in the present study.

**Fig 5:**
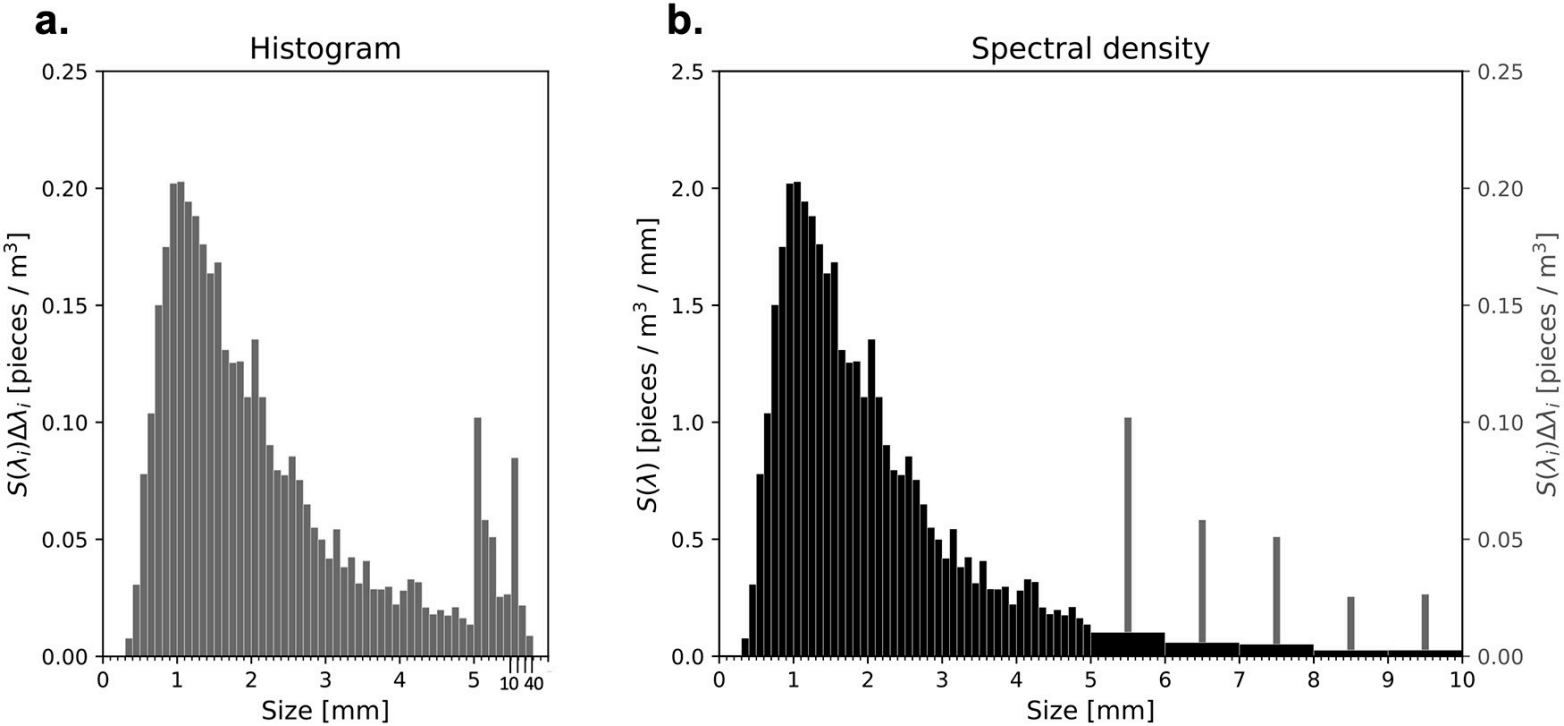
Size distribution expressed as a histogram (a) and as a spectral density (b) from Isobe et al’s [21] observation around Japan. The histogram is a replot of Isobe et al’s Fig 2. We have obtained the data by digitizing the original figure using WebPlotDigitizer version 4.3 (https://automeris.io/WebPlotDigitizer/). The size spectral density is indicated by black bars with its scale shown on the left axis of panel (b), and the gray bars on panel (b) are the same histogram with its scale on the right axis. The spectral density is plotted in such a way that the black bars exactly coincide with the gray ones for λ < 5 mm. In panel (b), sizes larger than 10 mm are omitted because the spectral values are almost zero there.

Fig 5a replots one of these size distributions as an example. As stated above, the bin width is not uniform: for this particualr data, Δλ = 0.1 mm for λ < 5 mm, Δλ = 1 mm for 5 mm < λ < 10 mm, and Δλ = 10 mm for λ > 10 mm. This is the reason that the concentration jumps up beyond λ > 5 mm. For comparison with theories, we introduce a “size spectral density” *n*_*i*_ (Fig 5b) such that

$$\begin{array}{c} \Delta \lambda_{i}\;n_{i}=N_{i}, \end{array}$$ (7)

where Δλ_*i*_ is the width of the *i*-th bin and *N*_*i*_ is Isobe et al.’s value for the bin. By this definition, each rectangular area of the spectral plot is proportional to the number of plastic fragments within the bin. The spectral density is less sensitive to the bin widths, and in the limit of Δλ → 0, it converges to a continuous size spectrum *S*(λ) such that $\int_{\lambda_{a}}^{\lambda_{b}}d\lambda \;S\left(\lambda \right)$ is the number of fragments between λ_*a*_ and λ_*b*_. All of Isobe et al’s size distributions are converted to size spectral densities in the present study.

### Observed data in Cózar et al

The plastic samples summarized in Cózar et al [20] were collected in the accumulation zones [56] around the world (Fig 4a) from December 2010 to July 2011. Their method of sampling was the same as that of Isobe et al’s studies, except for the different mesh size (0.2 mm at the minimum) and mouth area (1 × 0.5 m^2^) of the neuston net and the different towing durations (10–15 min). The collected plastic fragments are classified into bins whose widths increase exponentially (like Δλ_*k*_ = *c*10^0.1*k*^ mm, where *k* is the bin number) for 0.2 mm < λ < 100 mm.

Cózar et al summed the number of plastic pieces over each accumulation zone without dividing the number by the volume of the sampled sea water (S6 Fig in their paper). Their data are digitized and converted to spectral densities in the same way as described above for Isobe et al’s data. Sizes larger than 40 mm in the tail of the distribution have had to be omitted because the numbers are so small there that the data points are too close to the horizontal axis of the plots for the digitizer to resolve. This limitation does not significantly influence the curve fitting because the fitting result is most highly sensitive to the values of the size spectral density around the peak size. The present study has also digitized Cózar et al’s S10b Fig to create a logarithmic plot of the sum of the size distributions for all the observed accumulation zones (Fig 4d).

### Wave energy in source region

We use the Lebreton et al’s plastic dispersal simulation result [55] to identify the source regions of the plastic fragments sampled in the Cózar et al’s accumulation zones. Lebrenton et al’s numerical simulation provides the dispersion of virtual particles representing a set of plastic fragments originating from land following the sea-surface currents reproduced by an oceanic circulation modelling system HYCOM/NCODA [88]. The particles are released at coastal locations on the basis of two scenarios, the amount of released particles determined as a function of impervious surface area (ISA-based scenario) and of coastal population density (PD-based scenario), respectively. The former is intended to reflect contributions from major rivers and the latter from large cities. Contribution in percentage of each emission region to the amount of the plastics in the accumulation zones are summarized in Table 1 from Lebreton et al’s supplementary data. For example, the plastic emission from Australia accounts for about 9.5% of the amount of plastics found in the South Pacific accumulation zone for the ISA scenario.

Next, we estimate wave energy level (no units) at each emission region from the 6-grade wave energy levels along the global coastlines provided by Fairley et al [57]. Each of Lebreton et al’s emission regions for each emission scenario consists of multiple emission points (their S2–S7 Figs). We select the point with the largest emission within the region, look up Fairley et al’s figure to determine the wave energy level of the point, and assign the energy level to the region. If there are multiple points with similarly large energy levels, we assign the average energy level to the region. These energy levels are summarized in the “Energy Level” column of Table 1; the left and right subcolumns are for the ISA and PD senarios, respectively. Some of the energy level values are missing in the table because the selected emission points are not covered in Fairley et al’s map. Finally, the expected wave energy level that the plastics found in the accumulation zone experienced for each scenario is calculated by the contribution-weighted average ∑_*i*_ *E*_*i*_ *f*_*i*_/∑_*i*_ *f*_*i*_, where *E*_*i*_ and *f*_*i*_ denote the wave energy level and the contribution of the *i*-th source region, respectively. For the North Pacific, we calculate the expected wave energy level without contribution from China, assuming that plastic wastes from China go to the North Pacific accumulation zone via Japan. The resultant expected wave energy level for each accumulation zone is shown in S1 Fig in S1 File.

## Supporting information

S1 File (PDF)

S1 Data Minimal data set for figures and tables. (ZIP)

## References

[1] Jambeck JR, Geyer R, Wilcox C, Siegler TR, Perryman M, Andrady A, et al. Plastic waste inputs from land into the ocean. Science. 2015;347(6223):768–771. doi: 10.1126/science.1260352 25678662

[2] Barnes DK, Galgani F, Thompson RC, Barlaz M. Accumulation and fragmentation of plastic debris in global environments. Philos Trans R Soc B. 2009;364(1526):1985–1998. doi: 10.1098/rstb.2008.0205 PMC2873009 19528051

[3] Corcoran PL, Biesinger MC, Grifi M. Plastics and beaches: a degrading relationship. Mar Pollut Bull. 2009;58(1):80–84. doi: 10.1016/j.marpolbul.2008.08.022 18834997

[4] Andrady AL. Microplastics in the marine environment. Mar Pollut Bull. 2011;62(8):1596–1605. doi: 10.1016/j.marpolbul.2011.05.030 21742351

[5] Andrady AL. The plastic in microplastics: A review. Mar Pollut Bull. 2017;119(1):12–22. doi: 10.1016/j.marpolbul.2017.01.082 28449819

[6] Eriksen M, Lebreton LC, Carson HS, Thiel M, Moore CJ, Borerro JC, et al. Plastic pollution in the world’s oceans: more than 5 trillion plastic pieces weighing over 250,000 tons afloat at sea. PLoS ONE. 2014;9(12):e111913. doi: 10.1371/journal.pone.0111913 25494041 PMC4262196

[7] Van Sebille E, Wilcox C, Lebreton L, Maximenko N, Hardesty BD, Van Franeker JA, et al. A global inventory of small floating plastic debris. Env Res Lett. 2015;10(12):124006.

[8] Isobe A, Iwasaki S, Uchida K, Tokai T. Abundance of non-conservative microplastics in the upper ocean from 1957 to 2066. Nat Commun. 2019;10(1):1–13. doi: 10.1038/s41467-019-08316-9 30679437 PMC6345988

[9] Onink V, Wichmann D, Delandmeter P, Van Sebille E. The role of Ekman currents, geostrophy, and Stokes drift in the accumulation of floating microplastic. J Geophys Res: Oceans. 2019;124(3):1474–1490. doi: 10.1029/2018JC014547 31218155 PMC6559306

[10] Ashton K, Holmes L, Turner A. Association of metals with plastic production pellets in the marine environment. Mar Pollut Bull. 2010;60(11):2050–2055. doi: 10.1016/j.marpolbul.2010.07.014 20696443

[11] Holmes LA, Turner A, Thompson RC. Adsorption of trace metals to plastic resin pellets in the marine environment. Environ Pollut. 2012;160:42–48. doi: 10.1016/j.envpol.2011.08.052 22035924

[12] Nakashima E, Isobe A, Kako S, Itai T, Takahashi S. Quantification of toxic metals derived from macroplastic litter on Ookushi Beach, Japan. Environ Sci Technol. 2012;46(18):10099–10105. doi: 10.1021/es301362g 22916725

[13] Browne MA, Dissanayake A, Galloway TS, Lowe DM, Thompson RC. Ingested microscopic plastic translocates to the circulatory system of the mussel, Mytilus edulis (L.). Environ Sci Technol. 2008;42(13):5026–5031. doi: 10.1021/es800249a 18678044

[14] Boerger CM, Lattin GL, Moore SL, Moore CJ. Plastic ingestion by planktivorous fishes in the North Pacific Central Gyre. Mar Pollut Bull. 2010;60(12):2275–2278. doi: 10.1016/j.marpolbul.2010.08.007 21067782

[15] Murray F, Cowie PR. Plastic contamination in the decapod crustacean Nephrops norvegicus (Linnaeus, 1758). Mar Pollut Bull. 2011;62(6):1207–1217. doi: 10.1016/j.marpolbul.2011.03.032 21497854

[16] Jabeen K, Su L, Li J, Yang D, Tong C, Mu J, et al. Microplastics and mesoplastics in fish from coastal and fresh waters of China. Env Res Lett. 2017;221:141–149. 27939629 10.1016/j.envpol.2016.11.055

[17] Iwasaki S, Isobe A, Kako S, Uchida K, Tokai T. Fate of microplastics and mesoplastics carried by surface currents and wind waves: A numerical model approach in the Sea of Japan. Mar Pollut Bull. 2017;121(1-2):85–96. doi: 10.1016/j.marpolbul.2017.05.057 28559056

[18] Sagawa N, Kawaai K, Hinata H. Abundance and size of microplastics in a coastal sea: Comparison among bottom sediment, beach sediment, and surface water. Mar Pollut Bull. 2018;133:532–542. doi: 10.1016/j.marpolbul.2018.05.036 30041347

[19] Isobe A, Kubo K, Tamura Y, Nakashima E, Fujii N, et al. Selective transport of microplastics and mesoplastics by drifting in coastal waters. Mar Pollut Bull. 2014;89(1-2):324–330. doi: 10.1016/j.marpolbul.2014.09.041 25287228

[20] Cózar A, Echevarráa F, González-Gordillo JI, Irigoien X, Úbeda B, Hernández-León S, et al. Plastic debris in the open ocean. Proc Natl Acad Sci USA. 2014;111(28):10239–10244. doi: 10.1073/pnas.1314705111 24982135 PMC4104848

[21] Isobe A, Uchida K, Tokai T, Iwasaki S. East Asian seas: a hot spot of pelagic microplastics. Mar Pollut Bull. 2015;101(2):618–623. doi: 10.1016/j.marpolbul.2015.10.042 26522164

[22] Eo S, Hong SH, Song YK, Lee J, Lee J, Shim WJ. Abundance, composition, and distribution of microplastics larger than 20 *μ*m in sand beaches of South Korea. Environ Pollut. 2018;238:894–902. doi: 10.1016/j.envpol.2018.03.096 29631234

[23] Reisser JW, Slat B, Noble KD, Plessis KD, Epp M, Proietti MC, et al. The vertical distribution of buoyant plastics at sea: an observationalstudy in the North Atlantic Gyre. Biogeosciences. 2015;12:1249–1256. doi: 10.5194/bg-12-1249-2015

[24] Enders K, Lenz R, Colin Stedmon A, Torkel Nielsen G. Abundance, size and polymer composition of marine microplastics 10 *μ*m in the Atlantic Ocean and their modelled vertical distribution. Mar Pollut Bull. 2015;100(1):70–81. doi: 10.1016/j.marpolbul.2015.09.027 26454631

[25] Kooi M, Koelmans AA. Simplifying microplastic via continuous probability distributions for size, shape, and density. Env Res Lett. 2019;6(9):551–557.

[26] Lindeque PK, Cole M, Coppock RL, Lewis CN, Miller RZ, Watts AJ, et al. Are we underestimating microplastic abundance in the marine environment? A comparison of microplastic capture with nets of different mesh-size. Environ Pollut. 2020;265:114721. doi: 10.1016/j.envpol.2020.114721 32806407

[27] Tokai T, Uchida K, Kuroda M, Isobe A. Mesh selectivity of neuston nets for microplastics. Mar Pollut Bull. 2021;165:112111. doi: 10.1016/j.marpolbul.2021.112111 33588104

[28] Dohnanyi JS. Collisional model of asteroids and their debris. Journal of Geophysical Research. 1969;74(10):2531–2554. doi: 10.1029/JB074i010p02531

[29] Davis DR, Weidenschilling SJ, Farinella P, Paolicchi P, Binzel RP. Asteroid collisional history: Effects on sizes and spins. In: Binzel R, Gehrels T, Matthews MS, editors. Asteroids II. Univ. of Arizona Press, Tucson.; 1989. p. 805–826.

[30] Tanaka H, Inaba S, Nakazawa K. Steady-state size distribution for the self-similar collision cascade. Icarus. 1996;123(2):450–455. doi: 10.1006/icar.1996.0170

[31] Kolmogorov A. On the logarithmic normal distribution of particle sizes under grinding. In: Dokl. Akad. Nauk SSSR. vol. 31; 1941. p. 99–101.

[32] Middleton G. Generation of the log-normal frequency distribution in sediments. In: Topics in mathematical geology. Springer; 1970. p. 34–42. doi: 10.1007/978-1-4899-2708-8_4

[33] Crow EL, Shimizu K. Lognormal Distributions: Theory and Applications. Marcel Dekker, New York; 1988.

[34] Vincent P. Differentiation of modern beach and coastal dune sands—a logistic regression approach using the parameters of the hyperbolic function. Sedimentary Geology. 1986;49(3):167–176. doi: 10.1016/0037-0738(86)90036-9

[35] Kobayashi N, Kohyama K, Sasaki Y, Matsushita M. Statistical laws for food fragmentation by human mastication. J Phys Soc Japan. 2006;75(8):083001. doi: 10.1143/JPSJ.75.083001

[36] Ishii T, Matsushita M. Fragmentation of Long Thin Glass Rods. J Phys Soc Japan. 1992;61(10):3474–3477. doi: 10.1143/JPSJ.61.3474

[37] Weibull W. Wide applicability. Journal of applied mechanics. 1951;103(730):293–297. doi: 10.1115/1.4010337

[38] Brown WK, Wohletz KH. Derivation of the Weibull distribution based on physical principles and its connection to the Rosin–Rammler and lognormal distributions. J Appl Phys. 1995;78(4):2758–2763. doi: 10.1063/1.360073

[39] Poulain M, Mercier MJ, Brach L, Martignac M, Routaboul C, Perez E, et al. Small microplastics as a main contributor to plastic mass balance in the North Atlantic subtropical gyre. Environ Sci Technol. 2018;53(3):1157–1164. doi: 10.1021/acs.est.8b05458 30575384

[40] Pabortsava K, Lampitt RS. High concentrations of plastic hidden beneath the surface of the Atlantic Ocean. Nat Commun. 2020;11(1). doi: 10.1038/s41467-020-17932-9 32811835 PMC7434887

[41] Anderson TL. Fracture mechanics: fundamentals and applications. CRC press; 2017.

[42] Biswas S, Ray P, Chakrabarti BK. Statistical physics of fracture, breakdown, and earthquake: effects of disorder and heterogeneity. John Wiley & Sons; 2015.

[43] Irwin GR. Analysis of stresses and strains near the end of a crack traversing a plate. J Appl Mech. 1957;. doi: 10.1115/1.4011547

[44] Rice JR. A path independent integral and the approximate analysis of strain concentration by notches and cracks. J Appl Mech. 1968;. doi: 10.1115/1.3601206

[45] Rice JR, Rosengren GF. Plane strain deformation near a crack tip in a power-law hardening material. J Mech Phys Solids. 1968;16(1):1–12. doi: 10.1016/0022-5096(68)90013-6

[46] de Vladar HP, Barton NH. The contribution of statistical physics to evolutionary biology. Trends Ecol Evol. 2011;26(8):424–432. doi: 10.1016/j.tree.2011.04.002 21571390

[47] Bouchet F, Venaille A. Statistical mechanics of two-dimensional and geophysical flows. Phy Rep. 2012;515(5):227–295. doi: 10.1016/j.physrep.2012.02.001

[48] Vallianatos F, Papadakis G, Michas G. Generalized statistical mechanics approaches to earthquakes and tectonics. Proc Math Phys Eng Sci. 2016;472(2196):20160497. doi: 10.1098/rspa.2016.0497 28119548 PMC5247524

[49] Wu W, McFarquhar GM. Statistical Theory on the Functional Form of Cloud Particle Size Distributions. J Atmos Sci. 2018;75(8):2801–2814. doi: 10.1175/JAS-D-17-0164.1

[50] Kittel C, Kroemer H. Thermal physics. 2nd ed. W. H. Freeman and Company; 1980.

[51] Holthuijsen LH. Waves in Oceanic and Coastal Waters. Cambridge University Press; 2007. Available from: https://doi.org/10.1017%2Fcbo9780511618536.

[52] Tuomi L, Kahma KK, Pettersson H. Wave hindcast statistics in the seasonally ice-covered Baltic Sea. Boreal Env Res. 2011;16:451–472.

[53] Haselsteiner AF, Thoben KD. Predicting wave heights for marine design by prioritizing extreme events in a global model. Renew Energ. 2020;156:1146–1157. doi: 10.1016/j.renene.2020.04.112

[54] Met Office. Cartopy: a cartographic python library with a Matplotlib interface; 2010–2015. Available from: https://scitools.org.uk/cartopy.

[55] Lebreton LCM, Greer SD, Borrero JC. Numerical modelling of floating debris in the world’s oceans. Mar Pollut Bull. 2012;64(3):653–661. doi: 10.1016/j.marpolbul.2011.10.027 22264500

[56] Maximenko N, Hafner J, Niiler P. Pathways of marine debris derived from trajectories of Lagrangian drifters. Mar Pollut Bull. 2012;65(1-3):51–62. doi: 10.1016/j.marpolbul.2011.04.016 21696778

[57] Fairley I, Lewis M, Robertson B, Hemer M, Masters I, Horrillo-Caraballo J, et al. A classification system for global wave energy resources based on multivariate clustering. Appl Energy. 2020;262:114515. doi: 10.1016/j.apenergy.2020.114515

[58] Takeoka H, Akiyama H, Kikuchi T. The Kyucho in the Bungo Channel, Japan—Periodic intrusion of oceanic warm water. J Oceanogr. 1993;49(4):369–382. doi: 10.1007/BF02234954

[59] Isobe A, Guo X, Takeoka H. Hindcast and predictability of sporadic Kuroshio-water intrusion (kyucho in the Bungo Channel) into the shelf and coastal waters. J Geophys Res: Oceans. 2010;115(C4).

[60] Nagai T, Hibiya T. Numerical simulation of tidally induced eddies in the Bungo Channel: A possible role for sporadic Kuroshio-water intrusion (kyucho). J Oceanogr. 2012;68(5):797–806. doi: 10.1007/s10872-012-0141-9

[61] Koelmans AA, Kooi M, Law KL, Van Sebille E. All is not lost: deriving a top-down mass budget of plastic at sea. Env Res Lett. 2017;12(11):114028. doi: 10.1088/1748-9326/aa9500

[62] Rummel CD, Jahnke A, Gorokhova E, Kühnel D, Schmitt-Jansen M. Impacts of biofilm formation on the fate and potential effects of microplastic in the aquatic environment. Environ Sci Technol Lett. 2017;4(7):258–267. doi: 10.1021/acs.estlett.7b00164

[63] Kooi M, Nes EHv, Scheffer M, Koelmans AA. Ups and downs in the ocean: effects of biofouling on vertical transport of microplastics. Environ Sci Technol. 2017;51(14):7963–7971. doi: 10.1021/acs.est.6b04702 28613852 PMC6150669

[64] Van Melkebeke M, Janssen C, De Meester S. Characteristics and sinking behavior of typical microplastics including the potential effect of biofouling: implications for remediation. Environ Sci Technol. 2020;54(14):8668–8680. doi: 10.1021/acs.est.9b07378 32551546

[65] Kukulka T, Proskurowski G, Morét-Ferguson S, Meyer D, Law K. The effect of wind mixing on the vertical distribution of buoyant plastic debris. grl. 2012;39(7). doi: 10.1029/2012GL051116

[66] Berk Z. Food process engineering and technology. Academic press; 2018.

[67] Duroudier JP. Size Reduction of Divided Solids. Elsevier; 2016.

[68] Blanc N, Mayer-Laigle C, Frank X, Radjai F, Delenne JY. Evolution of grinding energy and particle size during dry ball-milling of silica sand. Powder Technol. 2020;376:661–667. doi: 10.1016/j.powtec.2020.08.048

[69] Timér G, Blömer J, Kun F, Herrmann HJ. New universality class for the fragmentation of plastic materials. Phys Rev Lett. 2010;104(9):095502. doi: 10.1103/PhysRevLett.104.095502 20366993

[70] Kishimura H, Noguchi D, Preechasupanya W, Matsumoto H. Impact fragmentation of polyurethane and polypropylene cylinder. Physica A. 2013;392(22):5574–5580. doi: 10.1016/j.physa.2013.07.033

[71] Okabe A, Boots B, Sugihara K, Chiu SN. Spatial tessellations: concepts and applications of Voronoi diagrams. vol. 501. John Wiley & Sons; 2009.

[72] Bishop JE. Simulating the pervasive fracture and fragmentation of materials and structures using randomly close-packed Voronoi tessellations. Citeseer; 2008.

[73] Bishop JE, Martinez MJ, Newell P. Simulating fragmentation and fluid-induced fracture in disordered media using random finite-element meshes. Int J Multiscale Comput Eng. 2016;14(4). doi: 10.1615/IntJMultCompEng.2016016908

[74] Pourmoghaddam N, Kraus MA, Schneider J, Siebert G. The geometrical properties of random 2D Voronoi tesselations for the prediction of the tempered glass fracture pattern. ce/papers. 2018;2(5-6):325–339. doi: 10.1002/cepa.934

[75] Pourmoghaddam N, Kraus M, Schneider J, Siebert G. Relationship between strain energy and fracture pattern morphology of thermally tempered glass for the prediction of the 2D macro-scale fragmentation of glass. Glass Struct Eng. 2019;4(2):257–275. doi: 10.1007/s40940-018-00091-1

[76] Wang Z, Yang S, Li L, Tang Y, Xu G. A 3D Voronoi clump based model for simulating failure behavior of brittle rock. Eng Fract Mech. 2021;248:107720. doi: 10.1016/j.engfracmech.2021.107720

[77] Carpinteri A, Pugno N. Fractal fragmentation theory for shape effects of quasi-brittle materials in compression. Mag Concr Res. 2002;54(6):473–480. doi: 10.1680/macr.2002.54.6.473

[78] Carpinteri A, Cornetti P, Puzzi S. Scaling Laws and Multiscale Approach in the Mechanics of Heterogeneous and Disordered Materials. Appl Mech Rev. 2006;59(5):283–305. doi: 10.1115/1.2204076

[79] Weiss J. Fracture and fragmentation of ice: a fractal analysis of scale invariance. Eng Fract Mech. 2001;68(17-18):1975–2012. doi: 10.1016/S0013-7944(01)00034-0

[80] Englman R, Rivier N, Jaeger Z. Size-distribution in sudden breakage by the use of entropy maximization. J Appl Phys. 1988;63(9):4766–4768. doi: 10.1063/1.340114

[81] Nagahama H; Elsevier. Fractal fragment size distribution for brittle rocks. Int J Rock Mech Min Sci. 1993;30(4):469–471. doi: 10.1016/0148-9062(93)91728-2

[82] Carpinteri A, Chiaia B, Cornetti P. A scale-invariant cohesive crack model for quasi-brittle materials. Eng Fract Mech. 2002;69(2):207–217. doi: 10.1016/S0013-7944(01)00085-6

[83] Carpinteri A, Lacidogna G, Pugno N. Scaling of energy dissipation in crushing and fragmentation: a fractal and statistical analysis based on particle size distribution. Int J Fract. 2004;129(2):131–139. doi: 10.1023/B:FRAC.0000045713.22994.f2

[84] Alava MJ, Nukala PKVV, Zapperi S. Statistical models of fracture. Adv Phys. 2006;55(3-4):349–476. doi: 10.1080/00018730300741518

[85] Wichmann D, Delandmeter P, van Sebille E. Influence of near-surface currents on the global dispersal of marine microplastic. J Geophys Res: Oceans. 2019;124(8):6086–6096. doi: 10.1029/2019JC015328 PMC6559306 31218155

[86] Butt HJ, Graf K, Kappl M. Physics and chemistry of interfaces. John Wiley & Sons; 2013.

[87] Bose SN. Planck’s law and light quantum hypothesis. Z Phys. 1924;26(1):178.

[88] Cummings JA. Operational multivariate ocean data assimilation. Quart J Royal Met Soc, Part C. 2005;131(613):3583–3604. doi: 10.1256/qj.05.105

