# Supplementary material for: A model for the size distribution of marine microplastics: A statistical mechanics approach
Source: PLoS One. 2021 Nov 30;16(11):e0259781. doi: 10.1371/journal.pone.0259781 (PMC8631679; doi:10.1371/journal.pone.0259781)
Supplement: S1 File — (PDF) [file pone.0259781.s001.pdf]

Supporting Information for
"A model for the size distribution of marine microplastics: a statistical mechanics approach"

Aoki and Furue

All the references cited here are included in the main manuscript with the same number.

S1 Appendix Derivation of total abundance

In this section, we calculate the total number and total mass of the plastic fragments. In the main text, the amplitude A is nondimensional when $S(\lambda)$ is fitted to an observed size spectrum per unit volume of sea water or it has the dimension of length cubed when the observation is a raw size spectrum as in Cózar et al. Accordingly, the following total number and mass are regarded as per unit volume of sea water or raw depending on which type of size spectrum $S(\lambda)$ denotes.

A transformation of variables $\nu' = \nu/\gamma^*$ in (6) leads to

$$S(\nu)d\nu = A\gamma^{*3}\nu'^2 \frac{1}{e^{\nu'}-1}d\nu'. \tag{S1}$$

The total number of plastic fragments over $0 < \lambda < \Lambda$ ($\leq L$) can then be written as

$$N \equiv \int_{1/\Lambda}^{\infty} S(\nu)d\nu = \int_{1/\gamma^*\Lambda}^{\infty} \frac{A\gamma^{*3}\nu'^2}{e^{\nu'}-1}d\nu' \tag{S2}$$

and with this familiar formula $(e^{\nu'}-1)^{-1} = \sum_{j=1}^{\infty} e^{-j\nu'}$,

$$\begin{aligned} N &= A\gamma^{*3}\sum_{j=1}^{\infty}\int_{1/\gamma^*\Lambda}^{\infty} \nu'^2 e^{-j\nu'}d\nu' \\ &= A\gamma^{*3}\left[2\,\mathrm{Li}_3(e^{-1/\gamma^*\Lambda}) + 2\left(\frac{1}{\gamma^*\Lambda}\right)\mathrm{Li}_2(e^{-1/\gamma^*\Lambda}) - \left(\frac{1}{\gamma^*\Lambda}\right)^2 \ln(1-e^{-1/\gamma^*\Lambda})\right], \end{aligned} \tag{S3a}$$

where

$$\mathrm{Li}_s(z) \equiv \sum_{j=1}^{\infty}\frac{z^j}{j^s}.$$

When $\Lambda \gg \gamma^{*-1}$,

$$N \approx A\gamma^{*3} 2\,\mathrm{Li}_3(1) = \sigma A\gamma^{*3}, \tag{S3b}$$

where $\sigma \equiv 2.404$, because $\mathrm{Li}_3(1) = \sum_{j=1}^{\infty} j^{-3} \simeq 1.202$ (known as Apéry's constant; see https://oeis.org/A002117). This approximation is equivalent to $\int_{1/L}^{\infty} S(\lambda)d\lambda \approx \int_0^{\infty} S(\lambda)d\lambda$. This approximation is a natural one when $\Lambda \sim L$ because $1/\gamma^*\Lambda \sim 1/\gamma^*L = 2L\Delta h\phi/\gamma$ and this factor is therefore the ratio of the surface energy $L\Delta h\phi$ to the mean environmental energy γ. We naturally assume that $L\Delta h\phi \ll \gamma$ because otherwise not many small fragments would be generated.

Similarly, the total mass of plastics is

$$M \equiv \int_0^{\Lambda} \rho\lambda^2\Delta h\, S(\lambda)d\lambda, \tag{S4}$$

where ρ is the mass density of the plastic material and Δh is the thickness of the original plate. After similar transformations as above,

$$M = A\gamma^*\rho\Delta h\sum_{j=1}^{\infty}\frac{e^{-j/\gamma^*\Lambda}}{j} = -A\gamma^*\rho\Delta h\ln(1-e^{-1/\gamma^*\Lambda}) \tag{S5a}$$

$$\approx A\gamma^*\rho\Delta h\ln(\gamma^*\Lambda), \tag{S5b}$$

using the same approximation, $\gamma^*\Lambda \gg 1$, as for N. Unlike N, M depends on Λ even when $\gamma^*\Lambda \gg 1$ because the contribution of larger plastic pieces is significant to M whereas it is negligible to N.

S2 Appendix Formal analogy with theoretical physics

Our size spectrum is derived in analogy with black body radiation. Here we outline the derivation of the wavenumber spectrum of black body radiation and discuss the analogy. Since the derivations of the Boltzmann distribution and Planck's spectrum below are standard, we do not cite references there. See the literature on statistical mechanics for details (e.g., Kittel and Kuroemer 1980 [50]).

Boltzmann distribution. Consider a large isolated system (heat bath) and a small subsystem, and classify the states which the subsystem can take by their energy value E. Assume that the subsystem takes a state with E at a probability of $p(E)$. Then we calculate the probability distribution that maximizes the entropy of the subsystem,

$$S = -k \int_0^{\infty} [p(E) \ln p(E)]\, \Omega(E)\, dE, \tag{S6}$$

where $\Omega(E)$, known as the "density of states," is the number of states with energy value E in the subsystem, under the constraints that $\int_0^{\infty} p(E)\, dE = 1$ and that the expected value of energy

$$\langle E \rangle = \int_0^{\infty} E\, p(E)\, \Omega(E)\, dE \tag{S7}$$

is given. The solution is

$$p(E) = \frac{e^{-\beta E}}{Z}, \qquad \text{where} \quad Z \equiv \int_0^{\infty} e^{-\beta E}\, \Omega(E)\, dE. \tag{S8}$$

The variable Z as a function of β is called the "partition function".

This probability distribution is known as the Boltzmann distribution. The variable β, which enters the solution because of the energy constraint, is related to the temperature of the system through the thermodynamic relation

$$\frac{1}{T} = \frac{\partial S}{\partial \langle E \rangle} = \beta k.$$

For the second equality, we have used (S6)–(S8) to calculate the derivative. We replace β with kT in what follows.

Black body radiation. Consider a mass of material, a "black body", which is in thermal equilibrium and assume that there is a vacuum cavity within it. Photons are emitted from the black body into the vacuum cavity and absorbed by the opposite wall. The energy of the photons obeys the energy probability distribution of the black body, which is assumed to be the Boltzmann distribution. Planck further assumed that the the energy of a photon with frequency ω can take only a value which is an integral multiple of the unit $\hbar\omega$, where $\hbar$ is a universal constant. The energy of the photons in the cavity, accordingly, takes the form of

$$\varepsilon(j, \omega) = j\hbar\omega. \tag{S9}$$

Since (S9) obeys the Boltzmann distribution, we can convert the probability of photon energy as a function of j with ω regarded as a parameter using (S9) and (S8). The result is $p(j;\omega) = e^{-jh\omega/kT} / \sum_j e^{-jh\omega/kT}$. In this case, the expected energy can be calculated as $\langle E \rangle = \sum_j j\hbar\omega\, p(j;\omega)$ and hence

$$\langle E \rangle(\omega) = \frac{\hbar\omega}{e^{\hbar\omega/kT} - 1} \tag{S10}$$

for each frequency. Also, this energy divided by $\hbar\omega$ provides the expected number of photons

$$\frac{1}{e^{\hbar\omega/kT}-1}. \tag{S11}$$

This formula is known as the Bose distribution.

Finally, we convert the energy distribution (S10) to the frequency spectrum of energy considering the dimensionality of the space. The frequency interval $(\omega, \omega + d\omega)$ includes $\omega^2/\pi^2 c^3$ modes of the wave per unit volume of the three-dimensional cavity. Since the energy spectrum is the product between the number of modes included in the interval $(\omega, \omega + d\omega)$ and the expected value of the energy at ω, which is given by (S10), the result is Planck's energy spectrum

$$\frac{\hbar}{\pi^2 c^3}\frac{\omega^3}{e^{\hbar\omega/kT}-1}. \tag{S12}$$

By analogy, the photons correspond to the fragmented plastic litters on beaches, and the black body to the environment (the weather and wave conditions) (Fig. A). The mean energy of the black body, kT, corresponds to the mean crush energy, γ, of the environment. Further, the crush energy is expressed as $\varepsilon = jb\nu$ (Eq. 3) on the basis of the necessary surface energy. This expression corresponds to (S9) in Planck's theory. Because of this analogy, the Boltzmann distribution (Eq. 4 with Eq. 3) and the Bose distribution (Eq. 5) in our model are formally the same as those of Planck's theory.

Our size spectrum (Eqs. 1 and 6) is analogous to Planck's (S12). As compared to (6), Planck's formula can be obtained formally if we take $A \to \hbar/\pi^2 c^3$ and $\gamma \to kT$, $\nu \to \omega$ after multiplying the right-hand side of (6) by ν. The last multiplication is merely due to the dimensionality of the space: the numerator of our formula becomes also proportional to ν^3 if the fragmentation of plastics is three-dimensional (see below). The corresponding wavelength (size) spectrum is obtained from the relation $\lambda = c/2\pi\omega$ for Planck's and $\lambda = 1/\nu$ for our spectrum.

The transition from (S10) to (S12) does not have a close analogy with the plastic model. As we have seen, Planck's energy distribution is essentially

$$(\text{number of modes} \propto \omega^2) \times (\text{energy of a photon } \hbar\omega) \times (\text{Bose distribution})$$

On the other hand, our plastic "wavenumber" spectrum is essentially

$$(\text{number of fragments} \propto \nu^2) \times (\text{Bose distribution}),$$

where the Bose distribution describes the expected number of original plastic pieces (plates) which are fragmented. If the original plastic piece is a cube and its fragmentation is three-dimensional, the number of fragments will be proportional to ν^3 and the functional form of the plastic "wavenumber" distribution with respect to ν will be exactly the same as Planck's (See S4 Appendix).

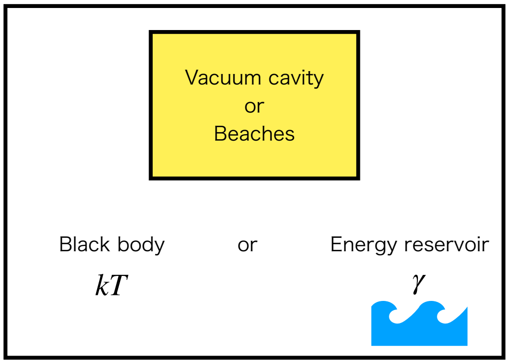

Fig. A. Analogy between black-body radiation and microplastics. The yellow area represents the vacuum cavity where electromagnetic radiation occurs or the beaches where microplastics are produced. The white area represents the blackbody characterized by kT, or the energy reservoir (winds, waves, and so on) characterized by γ.

S3 Appendix Superposition of size distributions

Size distribution. We explore how the size distribution is modified if multiple source regions with different parameters contribute. Here we assume that each source region contributes the same number of plastic fragments (N), which gives A in (1) as a function of γ^*:

$$A = \frac{N}{\sigma\gamma^{*3}} = \frac{N}{2.404\gamma^{*3}}$$

according to (2). In this case, the size spectrum (1) can be written as

$$S^*(\lambda; a, b^*) \equiv a\frac{b^{*3}}{\lambda^4}\frac{1}{e^{b^*/\lambda} - 1},$$

where a is a nondimensional constant and $b^* \equiv b/\gamma = 1/\gamma^*$.

We next calculate the average of $S^*(\lambda; 1, b^*)$ from $b_c^* - \Delta b^*/2$ to $b_c^* + \Delta b^*/2$. The order of magnitude of b^* is known because the value of λ that gives the peak of the size spectrum is $O(b^*)$ (it can be shown that it is approximately $0.255b^*$ from Eq. 1 and this λ value is constrained by observations). The average is calculated numerically changing b^* at an interval of 0.1 mm. For an illustration, and we look at three cases with $(b_c^*, \Delta b^*) = (4\,\text{mm}, 4\,\text{mm}), (7\,\text{mm}, 4\,\text{mm}), (5\,\text{mm}, 8\,\text{mm})$, and plot the results in Figs. Ba, Bc, and Be, respectively. The solid black curve plots the averaged S^*; the dashed and dotted curves plot $S^*(\lambda; 1, b_1^*)$ and $S^*(\lambda; 1, b_2^*)$, where $b_1^* \equiv b_c^* - \Delta b^*/2$ and $b_2^* \equiv b_c^* + \Delta b^*/2$.

We then fit $S^*(\lambda; a, b^*)$ to the average profile by adjusting a and b^*, which is the red curve. This is to simulate the fitting of our theoretical curve to an observation which may be a mixture of plastic pieces from different origins. Compared to the "pure" profile (red curve), the peak of the average profile (black curve) shifts leftward, the peak value is lower, and the values are larger in the smallest size range.

The right panels of Fig. B plot the error (cyan curve) of the fitting of the pure profile to the average as a function of Δb^* with the same b_c^* value as in the respective left panel, which corresponds to the maximum value of Δb^* of the right panel. As expected, the fitting error grows with Δb^*. The green curve plots the optimal b^* as a function of Δb^*. The optimal b^* changes little and stays close to b_c^* (thin gray line), indicating that the value of b^* ($= 1/\gamma^*$ by definition) obtained by fitting observations is close to its average value.

Fig. C plots the average and optimal profiles from Fig. Ba but with the horizontal axis logarithmic (panel a) and with both axes logarithmic (panel b). The difference between the two curves is qualitatively similar to the difference between the observed and the best-fit theoretical curves for Cózar et al's South Atlantic data in Fig. 4d.

Total mass. Here we explore the impacts of superposition on the total mass. Suppose that the observed size distribution is a superposition of different distributions with different values of A, γ, ϕ, L, and Δh (See Fig. 1). We denote those parameter values for each distribution by A_k, γ_k, etc. for $k = 1, \ldots, K$. Assume that the shape of the superposition is similar to a "pure" distribution as in Figs. Ba and Bc.

By fitting our model spectrum to the observed, we obtain optimal values for b^* and A. Because the size distribution is similar to the corresponding "pure" distribution, (S5a) or (S5b) should give an accurate total mass. In the main text, we used this approach to infer the value of Δh so that the calculated total mass agrees with the observed. This approach can be formulated by, if we use the approximate form (S5b) for simplicity,

$$\frac{\rho\Delta h A}{b^*}\ln(\Lambda/b^*) = \sum_{k=1}^{K}\frac{\rho_k A_k}{c_k}\ln\frac{\Lambda}{c_k\Delta h_k},$$

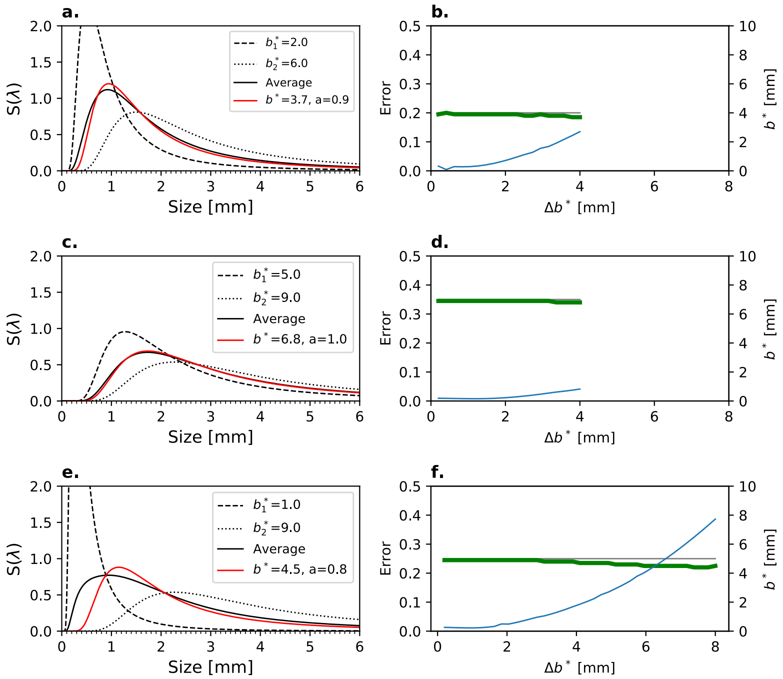

Fig. B. Superposition of size distributions with different values of b^* $(= 1/\gamma^*)$ ranging from $b_c^* - \Delta b^*/2$ to $b_c^* + \Delta b^*/2$ for **(a)** $b_c^* = 4\,\text{mm}$ and $\Delta b^* = 4\,\text{mm}$, **(c)** $b_c^* = 7\,\text{mm}$ and $\Delta b^* = 4\,\text{mm}$, and **(e)** $b_c^* = 5\,\text{mm}$ and $\Delta b^* = 8\,\text{mm}$. The dashed and dotted curves on the left panels are $S(\lambda; 1, b_1^*)$ and $S(\lambda; 1, b_2^*)$, respectively, where $b_{1,2}^* \equiv b_c^* \pm \Delta b^*/2$. The solid black curve is $S(\lambda; 1, b^*)$ averaged from b_1^* to b_2^*. The red curve indicates the best-fit size spectral density $S(\lambda; a_{\text{opt}}, b_{\text{opt}}^*)$ to the average. The right panels (**b,d,f**) show the fitting error (cyan) and the optimal b^* of the best-fit curve (green) as a function of Δb^*, where b_c^* and the maximum value of Δb^* are the same as in the corresponding left panel. The error is defined as the ratio of the norm of the difference between the average and best-fit curves to the norm of the average. The thin horizontal gray line denotes b_c^*.

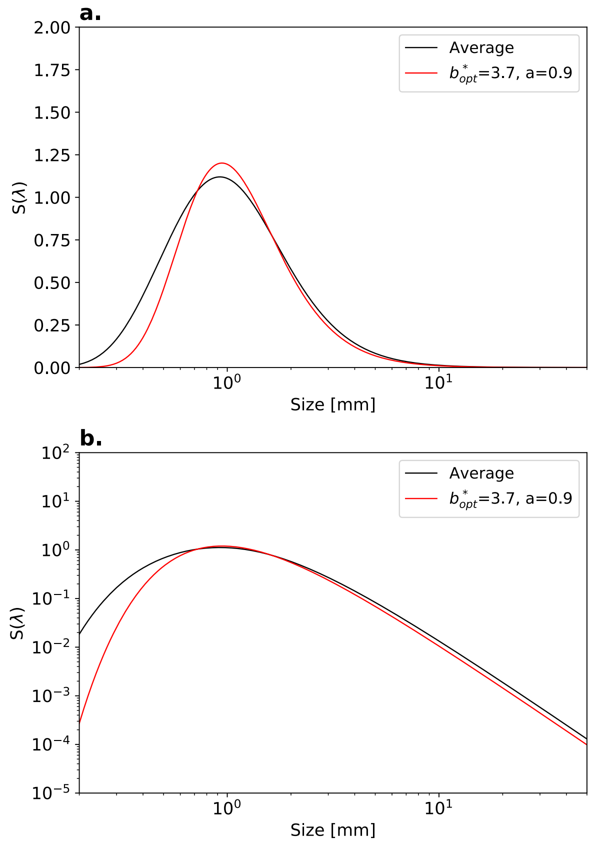

Fig. C. Size spectral density averaged over b^* (black) and the best-fit curve (red) with optimal values for b^* and a. Both curves are the same as the black and red curves of Fig. Ba except the horizontal axis (**a**) and both axes (**b**) are logarithmic in this figure.

where $c_k \equiv b_k^*/\Delta h_k = 2L_k^2\phi_k/\gamma_k$. The values of A and b^* on the left-hand side are those obtained by fitting the observed distribution and Δh on the left-hand side is the inferred value. Therefore, the inferred Δh is an "average" of Δh_k's in the sense that

$$\Delta h = \frac{b^*}{\rho A \ln(b^*/\Lambda)} \sum_{k=1}^{K} \frac{\rho_k A_k}{c_k} \ln \frac{c_k \Delta h_k}{\Lambda}.$$

Obviously, the result depends on the parameters A_k, γ_k, etc. If, for example, we assume that the each source contributes an equal number of plastic fragments (N), then $A_k = b_k^{*3} N/\sigma = (c_k \Delta h_k)^3 N/\sigma$, and the resultant dependency of the inferred Δh on Δh_k is

$$\Delta h = \frac{b^*}{\rho A \ln(b^*/\Lambda)} \sum_{k=1}^{K} \frac{\rho_k c_k^2 \Delta h_k^3 N}{\sigma} \ln \frac{c_k \Delta h_k}{\Lambda}.$$

S4 Appendix Three-dimensional model for fine microplastics

Our plate model presented in the Fracture model section and the Materials and Methods section (Fig. 1) implicitly allows for fragmented cells whose lateral size λ is smaller than the thickness Δh of the original plate. It is not very realistic to produce such small fragments from two dimensional fragmentation only and we do not attempt to apply our two-dimensional fracture model directly to plastic fragments for which $\lambda \ll \Delta h$. We instead construct a three-dimensional version of our model to explain fine microplastics ($\sim$10 µm$< \lambda < \sim$300 µm) recently observed in the upper ocean or on a beach [22, 24, 39, 40]

	$n = 2$	$n = 3$	$n = 4$
**Visual image**	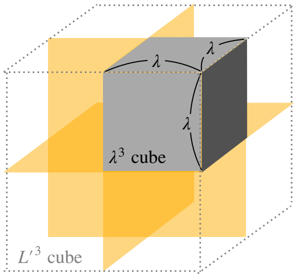	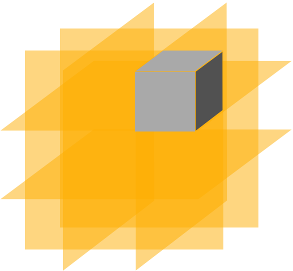	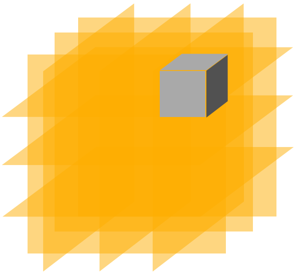
**Number of pieces** $n \times n \times n$	8	27	64
**Fractured surface area** $3(n-1)L'^2$	$3L'^2$	$6L'^2$	$9L'^2$

Fig. D. Schematic representation of 3-dimensional fracture model. All idealized plastic cube with a volume of L'^3 is broken into $n \times n \times n$ (middle) small cubes with the equal size of $\lambda = L'/n$. Orange planes show the surface created by the breakage and the total area of the planes depending on n is shown at the bottom.

Three-dimensional model. As an extension of the plate model, we consider the fracture of a cube with a volume of L'^3 into $n \times n \times n$ equal-sized cubic cells. Shcematics are shown in Figure D. The cell size is then $\lambda = L'/n$; with use of its inverse $\nu \equiv n/L'$, similarly to the original plate model, the number of pieces of the cells can be expressed as $L'^3\nu^3$. Also, the area of the new surfaces produced in this breakage is $3(n-1)L'^2$, which is proportional to n and hence to ν when $n \gg 1$. This allows defining the crush energy as $\varepsilon = jb\nu$, where j is the number of L'^3 cubes to be fractured and $b \equiv 3L'^3\phi$. Since this crush energy is formally the same as for the original plate model (3), the expected number of the fragments is given by the same Bose distribution as (5). Thus, the size spectrum for the 3-dimensional model can be expressed as

$$P(\nu)d\nu = A\nu^3 \frac{1}{e^{\nu/\gamma^*} - 1} d\nu \quad \text{or} \tag{S13}$$

$$S(\lambda)d\lambda = \frac{A}{\lambda^5} \frac{1}{e^{1/\lambda\gamma^*} - 1} d\lambda, \tag{S14}$$

where we have defined $\gamma^* \equiv \gamma/b$ as in the main manuscript. These formulae are the same as (6) and (1) except for the exponents on ν and λ, respectively. For a large size

limit, i.e., $\lambda\gamma^* \gg 1$, Eq. S14 asymptotes to $A\gamma^*/\lambda^4$. Also, the peak size is approximately $\lambda_p \simeq 0.201/\gamma^*$. In the similar fashion to the original plate model (See S1 Appendix), the total mass of the fragments in the size range from 0 to Λ ($\Lambda \leq L'$) is calculated as

$$M \equiv \int_0^\Lambda \rho\lambda^3 S(\lambda) d\lambda$$

$$= -\rho A\gamma^* \ln(1 - e^{-1/\gamma^*\Lambda}) \quad \text{(S15)}$$

$$\simeq \rho A\gamma^* \ln(\gamma^*\Lambda). \quad \text{(S16)}$$

Observed data. The 3-dimensional fracture model is applied to four observed size distributions of fine microplastics smaller than 300 µm. First, we compare with the size distribution obtained in the North Atlantic Ocean by Pabortsava and Lampitt [40] (PL2020). This data is the largest collection ($N = 1444$) of microplastics with sizes of 32 µm$< \lambda <$ 651 µm in the wide depth range from 10 m to 200 m. Since this collection, however, does not include any data near the sea surface, we also compare with the observed size distributions in the North Atlantic Ocean obtained by Enders et al [24] (EN2015) and Poulain et al [39] (PO2019) to complement this lack. The former collected finer microplastics with 11 µm$< \lambda <$ 300 µm sizes at ∼3 m depth ($N = 543$), and the latter those with 25 µm$< \lambda <$ 500 µm sizes in the water very close to the sea surface within 6 cm ($N = 520$). Further, we compare with the observed size distribution (20 µm$< \lambda <$ 1000 µm) obtained on Korean beach by Eo et al [22] ($N = 273738$), which could be considered as representative of the microplastics near the origin of their production. The size of the plastic pieces for all these observations is identified by the longest dimension. Additionally, EN2015 and PO2019 also use the geometric mean $\sqrt{d_L d_W}$, where d_L and d_W denote the length and width.

Application. The observed size distributions generally indicate an increase toward small size and sudden drop after passing the peak size for all data except for that in EN2015 (Fig. E). This feature is qualitatively similar to that of the microplastics collected using the neuston net with the mesh size of 300 µm (cf. Figs. 3 and 4). Using the optimal A and γ^* obtained by the least square method over the entire size range, the theoretical curves of the 3-dimensional fracture model are found to well match the observed size distributions. The optimal theoretical curve also reproduces the increase toward small size even for the size distribution in EN2015, which does not have a sudden drop. In this size range, the 3-dimensional fracture model seems to have higher reproducibility for PL2020 and EO2018 than the 2-dimensional model while the difference between the models is not clear for the other cases.

Note that we have chosen geometric mean for size in Figs. Ec and Eb. When plotted with longest dimension, they are respectively Figs. Fa and Fb. Our model fits better with geometric mean (Figs. Ec and Eb) in both cases. For EN2015, our model underestimates in the size range larger than 0.05 mm (Fig. Fa) and this tendency is weakened with geometric mean (Fig. Ec). That is, geometric mean shifts larger fragments leftward in this case. This is a natural consequence of geometric mean. For PO2019, geometric mean not only shifts the data leftward but also smooths the distribution. The distribution is still not quite smooth, suggesting that more samples would be needed to get a smooth distribution. Even with geometric mean, our model still underestimates for sizes larger than 0.05 mm for EN2015 (Figs. Ec and Fa). This might be because some of the samples were collected using a 50 µm (0.05 mm) mesh in some locations [24].

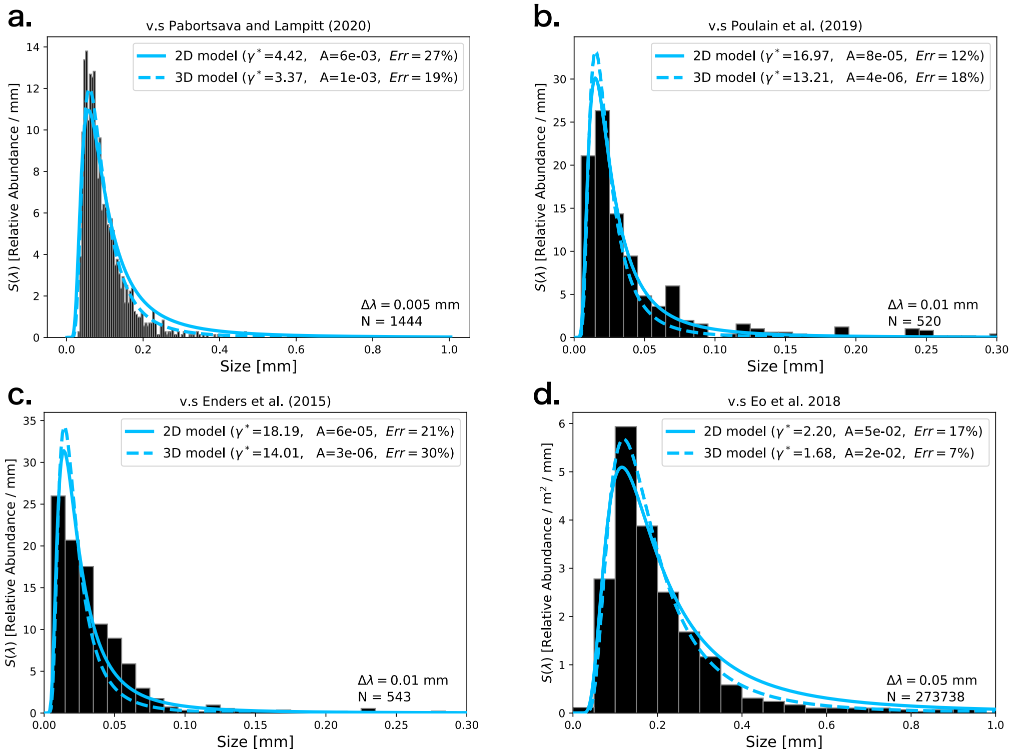

Fig. E. Size spectral densities of fine microplasitcs for observations (Black bars) and theories based on the original 2-dimensional plate model (Solid blue) and 3-dimensional model (Dashed blue). See Methods for conversion from histogram into size spectrum for the observed data. The observed data are obtained by digitizing the published original figures with WebPlotDigitizer (see the Materials and Methods section), except that the size distribution in **a** is constructed from the fragment size data. Pabortsava and Lampitt [40] plot their data separately for a few depth ranges and polymer types. We have downloaded their data and merged all the data without any weights to construct the spectrum. The spectrum is normalized by the total abundance N. The sizes of the collected fragments for **b** and **c** are defined as geometric mean (see "Observed data" in this Appendix).

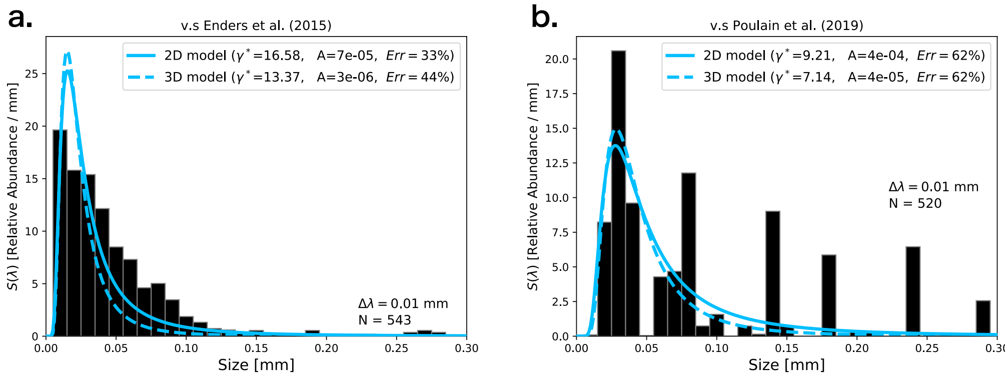

Fig. F. Panels **a** and **b** are respectively the same as Figs. Ec and Eb except that the size of collected fragments is defined as the longest dimension in these plots.

S5 Appendix Total length of the contact boundaries for fragments of irregular shape

Let us consider a square plastic specimen of size $L \times L$. As a first approximation, one could think the plate divided into $n \times n$ equal-sized pieces. In this case, the fragments have *regular* square shape, and the total length l of the contact boundaries is given by

$$l = 2\,(n-1)\,L = 2\,L^2\,\nu - 2\,L \tag{S17}$$

with ν being a wavenumber

$$\nu = \frac{n}{L} \tag{S18}$$

Equation (S17) can be rearranged in dimensionless form as

$$\frac{l}{2\,L} = L\,\nu - 1 \tag{S19}$$

Through numerical simulations, we will now show that equation (S19) is an excellent approximation for l when fragments have irregular shapes.

Firstly, we should define what we mean by "irregular". We notice that if we have a grid of points $\mathbf{x}_i = (x_i, y_i)\ \ i = 1, \ldots, n$, the resulting Voronoi tessellation [71] is a set of cells with regular shape (Fig. Ga), for which equation (S19) holds. We then perturb these positions

$$\tilde{x}_i = x_i + \Delta x_i \tag{S20}$$

$$\tilde{y}_i = y_i + \Delta y_i \tag{S21}$$

where

$$\Delta x_i = \alpha\, r_i\, \frac{1}{2\,\nu} \tag{S22}$$

and similarly for Δy_i, where $0 \leq r_i \leq 1$ is a random number drawn from a uniform distribution. The variable α defines the irregularity: when $\alpha = 0$, then $\tilde{x}_i = x_i$ and the fragments have all square shape.

With $\tilde{\mathbf{x}}_i = (\tilde{x}_i, \tilde{y}_i)$ now available, we construct a Voronoi tessellation clipped to the square of size $L \times L$. We then obtain $N \times N$ cells of irregular shape (figure Gb), where we can easily compute the perimeter l_i. The total length of the contact boundaries is then

$$l = \frac{1}{2}\left(\sum_i^N l_i - 4\,L\right) \tag{S23}$$

where the factor $1/2$ accounts for the double counting in the sum of all the l_i; the term $4\,L$ is necessary to subtract the perimeter of the square.

We carried out numerical simulations for $L = 100$ and $L = 150$, each time using $n = 10, 20, 30$ for a total of 6 cases. For each of these six cases, we ran 1000 simulations and computed the average l.

Figure H shows a comparison between regular and irregular (Voronoi) fragments. The total length l for the irregular shapes is very close to formula (S19), with a deviation of around 0.3%.

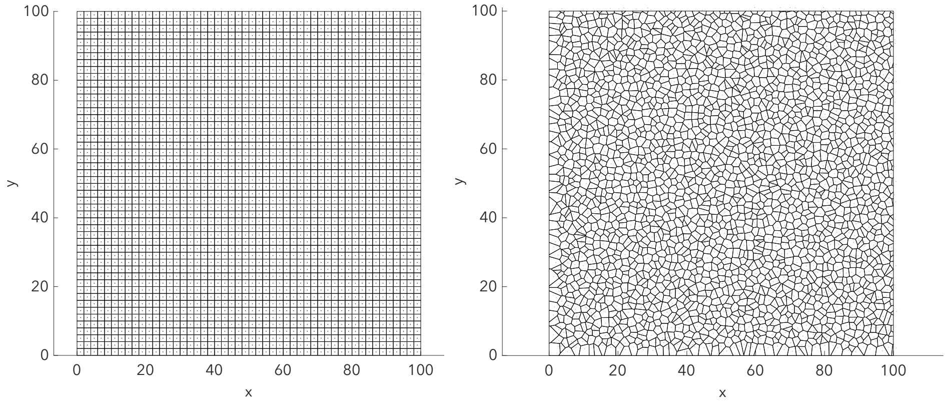

(a) $\alpha = 0$ **(b)** $\alpha = 2$

Fig. G. Voronoi tessellations representing fragments for a square patch of size $L = 100$; (a) regular and (b) irregular (b) arrangement of $n = 50$ points $\mathbf{x}_i$ (black dots).

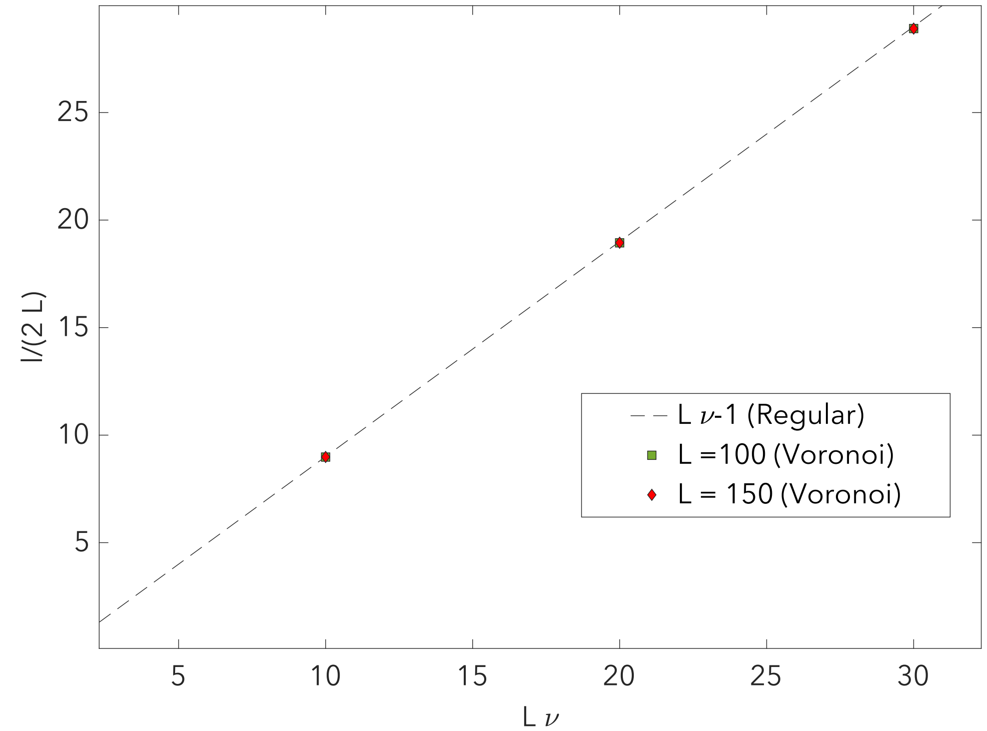

Fig. H. Equation (S19) for regular and irregular fragments.

S1 Figure

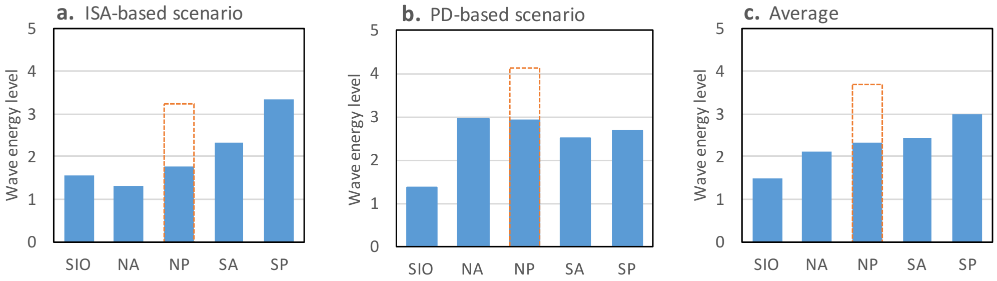

S1 Figure. Expected wave energy level (no units) for accumulation zone in Southern Indian Ocean (SIO), North Atlantic (NA), North Pacific (NP), South Atlantic (SA), and South Pacific (SP) (blue bars). Dashed orange bars denote the case without the contribution from China in the North Pacific accumulation zone.

S1 Table

S1 Table. Optimal γ^* for different observation regions

**Environmental energy γ^* [mm^{-1}]**	**Region**	**Literature**
0.24	North Atlantic Ocean	Cózar et al. 2014
0.24	Around Japan	Isobe et al. 2015
0.26	Western Pacific transoceanic section	Isobe et al. 2019
0.27	South Indian Ocean	Cózar et al. 2014
0.27	South Atlantic Ocean	Cózar et al. 2014
0.35	North Pacific Ocean	Cózar et al. 2014
0.35	South Pacific Ocean	Cózar et al. 2014
0.39	Seto Inland Sea	Isobe et al. 2014
